# Bundling actin filaments from membranes: some novel players

**DOI:** 10.3389/fpls.2012.00188

**Published:** 2012-08-24

**Authors:** Clément Thomas

**Affiliations:** Laboratory of Molecular and Cellular Oncology, Department of Oncology, Public Research Centre for Health (CRP-Santé)Luxembourg, Luxembourg

**Keywords:** actin bundling, fimbrins, formins, LIM proteins, SCAB1, THRUMIN1, V-ATPases, villins

## Abstract

Progress in live-cell imaging of the cytoskeleton has significantly extended our knowledge about the organization and dynamics of actin filaments near the plasma membrane of plant cells. Noticeably, two populations of filamentous structures can be distinguished. On the one hand, fine actin filaments which exhibit an extremely dynamic behavior basically characterized by fast polymerization and prolific severing events, a process referred to as actin stochastic dynamics. On the other hand, thick actin bundles which are composed of several filaments and which are comparatively more stable although they constantly remodel as well. There is evidence that the actin cytoskeleton plays critical roles in trafficking and signaling at both the cell cortex and organelle periphery but the exact contribution of actin bundles remains unclear. A common view is that actin bundles provide the long-distance tracks used by myosin motors to deliver their cargo to growing regions and accordingly play a particularly important role in cell polarization. However, several studies support that actin bundles are more than simple passive highways and display multiple and dynamic roles in the regulation of many processes, such as cell elongation, polar auxin transport, stomatal and chloroplast movement, and defense against pathogens. The list of identified plant actin-bundling proteins is ever expanding, supporting that plant cells shape structurally and functionally different actin bundles. Here I review the most recently characterized actin-bundling proteins, with a particular focus on those potentially relevant to membrane trafficking and/or signaling.

## Introduction

**Figure 1:**
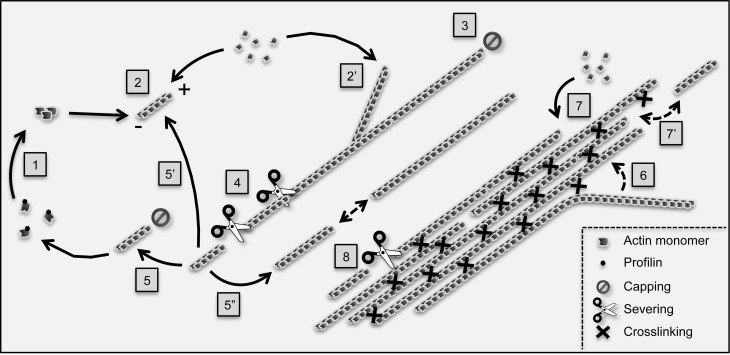
**Main reactions controlling actin filament dynamics and organization in plant cells.** The G-actin monomer binding protein profilin inhibits spontaneous actin nucleation in the cytoplasm. Nucleation is promoted *de novo* (1) by nucleating proteins such as formins. In addition, non-processive formins, such as *Arabidopsis* AtFH1, can also induce nucleation from the side of pre-existing filaments, a process which likely contributes to the initiation of actin bundles (not illustrated; Michelot et al., 2006; Blanchoin et al., 2010). Following nucleation, actin filaments undergo fast polymerization (2) and (2′) before being capped (3). The aging section of actin filaments (which contains ADP-loaded actin subunits, not shown) is fragmented by severing proteins such as actin-depolymerizing factors (4). The resulting fragments can be capped at their barbed end and depolymerize from their pointed (−) end to replenish the pool of monomers (5). Alternatively, they can re-elongate through polymerization (5′) although this process rarely occurs immediately following severing, suggesting intense barbed end capping activity (Staiger et al., 2009). Finally, actin fragments can serve as building blocks to assemble novel filaments by an end-joining mechanism (5″). Actin filaments are crosslinked into bundles by bundling proteins (right part of the cartoon). Both *in vitro* and live cell TIRFM-based analyses support that actin bundles form by a “catch and zipper” mechanism (6) (Khurana et al., 2010). Actin bundles subsequently grow by elongation of filaments at their ends (7) as well as by end-association of pre-existing filaments (7′), a process which might be facilitated by bundling proteins. Like single filaments, actin bundles are severed although at a lower frequency (Khurana et al., 2010; Smertenko et al., 2010). Current data support that unipolar bundles (here-exemplified) predominate in plant cells. However, the existence of bundles containing actin filaments of mixed polarity is not excluded.

Actin is one of the most abundant, ubiquitous, and conserved proteins in eukaryotes. In the cell, globular actin subunits polymerize into actin filaments which themselves assemble into higher order structures, such as orthogonal networks and parallel bundles (Figure 1). This system, referred to as the actin cytoskeleton, exhibits an extraordinary high degree of plasticity allowing the formation, destruction, and recycling of diverse filamentous structures within a short time scale, and offers countless possibilities to cells. The primary level of the regulation of actin cytoskeleton organization and dynamics consists in various (>100) actin-binding proteins which control, in time and space, actin filament nucleation, elongation, stabilization, capping, severing, and crosslinking (Pollard et al., 2000; Winder and Ayscough, 2005). In animal and yeast cells, cortical actin filaments and the plasma membrane undergo a dynamic interplay (Pollard and Cooper, 2009). For instance, the coordinated polymerization of actin filaments against the membrane provides the force necessary to modify cell shape and promote cell locomotion and division. As a consequence, dysfunctions in the actin polymerization machinery or in its regulation frequently results in diseases, including cancers (Van Troys et al., 2008a). Although related mechanisms are not excluded in plant cells, they are obviously not prevalent. The rigid plant cell wall precludes any modification of the cell boundary by an actin polymerization-based process. However, actin filaments are in close proximity with the cell membrane in plant cells too.

Recent studies combining advanced imaging approaches, such as variable-angle epifluorescence microscopy (VAEM), spinning disc confocal microscopy, and reliable fluorescent actin markers have advanced our understanding of the organization and dynamics of the actin cytoskeleton near the cell cortex (Staiger et al., 2009; Khurana et al., 2010; Smertenko et al., 2010; Henty et al., 2011; Wang et al., 2011; Toth et al., 2012; Figure 1). Cortical filaments arrange into complex networks whose stochastic behavior is largely consistent with the predictions of a biomimetic system (Michelot et al., 2007; Staiger et al., 2009; Blanchoin et al., 2010). Single filaments randomly polymerize at extremely high growth rates (1.7 μm/s in hypocotyl epidermal cells from *Arabidopsis* seedlings) and exhibit prominent buckling and straightening behavior. Most filaments are short-lived (<30 s), which was demonstrated to be primarily due to prolific severing activity rather than to filament end depolymerization. Beside single filaments, thicker and longer bundles adopt less convoluted configurations and tend to align with the long axis of the cell (Staiger et al., 2009; Smertenko et al., 2010; Henty et al., 2011). In comparison with finer filaments, thick fibers experience slower but qualitatively similar dynamics. Indeed, they elongate, buckle, bundle, and are severed (Staiger et al., 2009; Blanchoin et al., 2010; Smertenko et al., 2010). Although actin filament severing emerges as the leading driver of actin cytoskeleton remodeling, an additional mechanism involving filament bundling, unbundling, and myosin-dependent sliding events was suggested to contribute to the permanent reorganization of the cortical actin network (Smertenko et al., 2010). Using VAEM and quantitative approaches, Henty et al. (2011) provided, for the first time, direct evidence of the contribution of an ABP, namely *Arabidopsis* actin depolymerizing factor 4 (AtADF4), to actin stochastic dynamics *in vitro*. In agreement with the biochemical actin severing activity of AtADF4, hypocotyl epidermal cells from *adf4* knockout mutants exhibited a 2.5–3-fold decrease in the rate of severing, as well as increased filament lengths and lifetimes. The loss of AtADF4 also led to excessive actin bundling and cell growth in the apical region of the hypocotyl, where active cell expansion takes place.

Actin bundles have been repeatedly reported to play a critical role in cell morphogenesis (Baluska et al., 2001; Smith and Oppenheimer, 2005; Thomas et al., 2009; Higaki et al., 2010a). The relationships between the extent of actin bundling and cell growth are however complex as illustrated by contradictory observations. For instance, the growing epidermal cells from petioles of *adf4* mutants also exhibit enhanced bundling but, contrarily to hypocotyl epidermal cells, are shorter than in wildtype plants (Henty et al., 2011). Some studies have established that in specific cells, such as rice coleoptiles, increased actin bundling negatively impacts cell elongation (Nick et al., 2009; Nick, 2010). As an underlying mechanism, it has been proposed that actin bundling prevents efficient delivery of auxin-efflux carriers to their site of action at the plasma membrane, a process which would require a more unbundled actin configuration (Nick, 2010). Actin bundles most likely impact cell growth by different ways. Indeed, actin bundling can alter turgor pressure, the main physical driver of cell expansion (Szymanski and Cosgrove, 2009), by modifying the thickness of the cell wall and the shape of the transvacuolar strands and vacuole (e.g., Staiger et al., 1994; Higaki et al., 2010a,b, 2011). Finally, there is much evidence that actin bundles serve as main tracks used by myosin motors to drive endomembrane compartments over long distances to sites of growth (Smith and Oppenheimer, 2005; Thomas et al., 2009; Higaki et al., 2010a). Interestingly, the organization and dynamics of the actin cytoskeleton significantly differ in isotropically and anisotropically growing cells, and this was confirmed by recent quantitative analyses using VAEM (Smertenko et al., 2010). Therefore, the role of actin bundling in cell growth appears multiple and cell-type dependent.

In animal cells, the assembly of actin bundles is required for the formation and/or function of various specialized cellular structures, such as filopodia, lamellipodia, stress fibers, microvilli, and invadopodia (Stevenson et al., 2012). In most of these structures, actin bundles are in close connection with the cell membrane and, in some cases, with the extracellular environment referred to as the extracellular matrix. For instance, the α-actinin-induced actin bundles that constitute the ventral stress fibers of non-muscle cells are anchored to focal adhesions at each of their extremities. Focal adhesions transmit the force generated by myosin II-dependent stress fiber contraction to the extracellular matrix, allowing to pull the cell body during cell migration (Vicente-Manzanares et al., 2009; Ciobanasu et al., 2012). Key players of focal adhesions are the cell surface membrane receptors integrins around which assemble complex networks made of about 160 proteins that contribute to link the extracellular matrix to the actin cytoskeleton and to create a high-performance environmental sensing system (Geiger et al., 2009). Plants lack most focal adhesion components, including true integrin homologs. However, there is no doubt that plant cells perceive and transduce many external signals from their cell wall to their cytoskeleton (Baluska et al., 2003; Drobak et al., 2004; Humphrey et al., 2007; Fu, 2010; Higaki et al., 2011). Day et al. (2011) recently comprehensively reviewed the potential roles played by the actin cytoskeleton in the organization and activation of host responses to biotic stress. One of the earliest and well-documented responses of plant cells to fungal or oomycete pathogens is a reorganization of the actin cytoskeleton and endomembrane components which both focus at the site of infection (e.g., Kobayashi et al., 1994; Leckie et al., 1995; Xu et al., 1998; Opalski et al., 2005; Takemoto et al., 2006; Day et al., 2011). Such reorganization is thought to culminate in the formation of cell wall appositions rich in antimicrobial compounds (Hardham et al., 2007). During this process actin filaments become more bundled, suggesting an important role for actin-bundling proteins in the dynamic relocalization of organelles during interactions with pathogens. Interestingly, Hardham et al. (2008) could mimic pathogen-induced actin remodeling by applying a gentle and local pressure on the surface of *Arabidopsis* cotyledon epidermal cells, indicating that the actin cytoskeleton can readily reorganize (3–5 min after stimulation) in response to the physical force exerted by pathogens. Considering the continuous and fast remodeling of the cortical actin array observed in both growing and non-growing epidermal cells, an emerging and seducing idea is that cortical actin plays a sentinel role capable of initiating basal defense against pathogen-induced diseases or abiotic stress, such as mechanical stress, within short time scales (Staiger et al., 2009). How actin filaments and bundles communicate with the cell membrane and cell wall largely remains enigmatic.

Since our last review on actin bundling in plants (Thomas et al., 2009), more than fifteen additional plant actin-bundling proteins were isolated and characterized. Several of those belong to the previously known villin, formin, fimbrin, and LIM protein families, whereas others define novel families. Some of these proteins are likely direct linkers between actin bundles and the cell or organelle membranes. Here we review the last advances in plant actin-bundling proteins with a particular interest for those that further move ahead our comprehension about how actin bundles physically or functionally interact with membranes.

## Formins

Over the last years, formins have emerged as a large and major family of plant actin nucleating factors with critical functions in cell growth and division (Blanchoin and Staiger, 2008). Beside their core nucleating activity, plant formins display additional actin regulatory activities including nucleation, capping, severing, and bundling (Staiger and Blanchoin, 2006). The *Arabidopsis* formin AtFH1 (Banno and Chua, 2000), was the first plant formin reported to promote the formation of actin bundles both in live cells and *in vitro* (Cheung and Wu, 2004; Michelot et al., 2005). Its overexpression in pollen tubes stimulates the formation of actin bundles from the cell membrane and locally induces membrane deformation, suggesting that a proper density and distribution of actin bundles is critical for membrane assembly and/or maintenance, and that formins play substantial roles in these processes (Cheung and Wu, 2004). Mechanistic studies, employing total internal reflection fluorescence microscopy (TIRFM), revealed that AtFH1 functions as a non-processive formin which moves from the barbed end to the side of an actin filament after the nucleation event, and that this property is involved in AtFH1 actin bundling activity (Michelot et al., 2006). Recently, several other plant formins were shown to promote the formation of actin bundles in an autonomous manner, including *Arabidopsis* AtFH4, AtFH8, and AtFH14 (Deeks et al., 2010; Li et al., 2010; Xue et al., 2011), and rice OsFH5 (Yang et al., 2011; Zhang et al., 2011a).

Interaction of class I plant formins with a membrane is predicted by the characteristic membrane-targeting domain present in their N-terminal region which consists in a signal peptide followed by a transmembrane domain (Deeks et al., 2002; Blanchoin and Staiger, 2008). Accordingly, most class I formins examined so far were shown to accumulate at the cell periphery or in membrane-rich structures such as the cell plate using immunocytochemistry and/or GFP-fusion strategies (Cheung and Wu, 2004; Favery et al., 2004; Van Damme et al., 2004; Deeks et al., 2005; Ingouff et al., 2005; Cheung et al., 2010). Interestingly, AtFH8-GPF localizes primarily to the nuclear envelope in interphase cells, suggesting functional differences among class I formins (Xue et al., 2011). In addition, biochemical analyses indicate that pollen-specific AtFH3 lacks the ability to generate actin bundles *in vitro* (Ye et al., 2009). Nevertheless, with or without intrinsic actin-bundling activities, class I formins were convincingly demonstrated to promote actin bundling *in vivo* (Cheung and Wu, 2004; Ye et al., 2009; Cheung et al., 2010). Indeed, both gain- and loss-of-function genetic studies pointed out a central role of AtFH3 in regulating the long and thick actin bundles running along the pollen tube shank and in controlling the direction and velocity of cytoplasmic streaming (Ye et al., 2009). Therefore, AtFH3 likely cooperates with pollen actin-bundling proteins to assemble the tracks required for long distance actomyosin-dependent movement. Beside AtFH3, the pollen tube tip-enriched formin AtFH5 was found to play more specific functions in the formation of the subapical actin structure often referred to as the cortical actin fringe, and in membrane-targeted vesicular trafficking (Cheung et al., 2010).

It is noteworthy that class I formins exhibit divergent and potentially highly glycosylated extracellular domains, and accordingly represent excellent candidates for mediating extracellular stimuli to the actin cytoskeleton (Cvrckova, 2000; Blanchoin and Staiger, 2008), e.g., during the guidance of pollen tube growth in response to female tissue signals (Cheung and Wu, 2004). In this context, Martiniere et al. (2011) recently provided compelling evidence that the extracellular domain of AtFH1 is anchored to the cell wall and thereby reduces the lateral mobility of AtFH1. Domain analyses highlighted the central role in AtFH1 immobilization of a short, 15 amino acid-long, domain which includes a signature peptide of extensins, a class of cell wall-associated hydroxyproline-rich glycoproteins (Banno and Chua, 2000; Showalter et al., 2010). Although the biochemical nature of formin-cell wall interactions has not been resolved yet, it is tempting to propose that cell wall heterogeneity is responsible for targeting formins to specific plasma membrane subdomains. For instance, the accumulation of AtFH4 in cell-to-cell contact areas and of AtFH5 in the pollen-tube apical dome (Deeks and Hussey, 2005; Cheung et al., 2010) might reflect specificities in cell wall composition at these locations. Interestingly, the extracellular domain responsible for anchoring AtFH1 to the cell wall was required for AtFH1-mediated actin cytoskeleton remodeling in overexpression experiments (Martiniere et al., 2011). Although this remains speculative, anchoring of AtFH1 has been suggested to contribute to the formation and/or stabilization of AtFH1 functional dimers. Together these data support that AFH1, and most likely other class I formins, provide stable anchor points for the actin cytoskeleton at the cell membrane and can induce actin remodeling upon external signal perception. It is noteworthy that time lapse imaging analyses suggested that some of the AtFH5-nucleated and membrane-anchored actin filaments in the subapical region of pollen tubes are fragmented and released to the cytoplasm, providing precursors of some long actin bundles in the core cytoplasm (Cheung et al., 2010). Membrane-associated formins might therefore also indirectly contribute to the formation of more internal actin structures.

In addition to their function as interface between cell membrane and actin cytoskeleton, plant formins recently emerged as central links between actin filaments and microtubules. For instance, class I AtFH4, class II AtFH14, and the closely related rice OsFH5 bind to and bundle both actin filaments and microtubules and are accordingly expected to functionally coordinate the corresponding cytoskeletons (Deeks et al., 2010; Li et al., 2010; Yang et al., 2011; Zhang et al., 2011a). There is accumulating evidence that such coordination is crucial for many developmental processes such as intracellular transport, directional cell growth, and cell division (e.g., Fu et al., 2005; Collings, 2008; Wightman and Turner, 2008; Crowell et al., 2009; Petrasek and Schwarzerova, 2009). A recent quantitative study using VAEM revealed that microtubule depolymerization induces faster elongation and shortening of actin filaments, suggesting that actin dynamics at the cell cortex are modulated by microtubules (Smertenko et al., 2010). Although the underlying mechanism remains unknown, it seems reasonable to speculate that some formin family members are involved. Interestingly, endogenous OsFH5 localizes to specific regions at the chloroplast surface (Zhang et al., 2011a). Like other class II formins, OsFH5 possesses an N-terminal phosphate tensin (PTEN)-like domain instead of the typical transmembrane domain of most class I formins. Transient expression experiments indicate that the PTEN-like domain of FH5 is sufficient to target a fluorescent protein reporter to the chloroplast outer surface of tobacco cells, suggesting that it is responsible for the anchoring of OsFH5 to chloroplasts. Therefore OsFH5 emerges as a potential linker between actin filaments/bundles, microtubules, and chloroplasts, and might accordingly contribute to chloroplast motility, a process that has been proposed to rely on both cytoskeletons at least in some species (e.g., Chuong et al., 2006).

## Thrumin1

Chloroplasts change their subcellular location in response to light. They move toward weak light to optimize light capture for photosynthesis and away from intense light to minimize photodamage, the latter process being referred to as the avoidance response (Kasahara et al., 2002; Suetsugu and Wada, 2007). In plants, organelle movement primarily relies on class XI myosins which are predicted to transport their cargos along cytosplasmic actin bundles (Avisar et al., 2008; Peremyslov et al., 2008; Sparkes et al., 2008). Although myosin inhibitor studies support that chloroplast movement also depends on myosin activity to some extent (e.g., Paves and Truve, 2007), recent data indicate that chloroplasts primarily use another type of actin-based mechanism to rapidly change their direction in response to light. A population of so-called chloroplast actin filaments (cp-actin filaments) was shown to anchor chloroplasts to the plasma membrane suggesting that they are involved in light-induced chloroplast repositioning (Kadota et al., 2009; Suetsugu et al., 2010a,b). In this context, THRUMIN1 was recently identified as a novel actin-bundling protein with a potential critical role in linking phototropin photoreceptor activity at the plasma membrane and actin-dependent chloroplast movements (Whippo et al., 2011).

Compared to wild-type plants, *thrumin1* mutants exhibit slower and more randomized chloroplast movements in response to light stimuli. *In vitro* biochemical analyses indicate that THRUMIN1 binds to actin filaments in a direct manner and promotes the formation of actin bundles. Consistent with these data and the previously reported association of THRUMIN1 with the plasma membrane (Alexandersson et al., 2004), YFP-fused THRUMIN1 (THRUMIN1-YFP) extensively decorates the filamentous actin cytoskeleton along the plasma membrane, and in association with chloroplasts (Whippo et al., 2011). Upon stimulation of the chloroplast avoidance response by a localized blue-light irradiation, THRUMIN1-YFP further accumulates along actin filaments and apparently increases actin-bundling locally. The underlying mechanism was proven to be dependent on the phototropin blue-light photoreceptors PHOT1 and PHOT2. Indeed, no elevation of THRUMIN1-YFP fluorescence occurred in *phot1phot2* double mutants upon blue light stimulation. Together these data support that THRUMIN1 promotes the formation of actin bundles from the plasma membrane in response to light and in a phototropin-dependent manner. However, the exact role of such actin bundles in chloroplast movement remains to be established. In addition, how THRUMIN1 cooperates with CHUP1, a chloroplast outer envelope ABP involved in cp-actin filament formation (Oikawa et al., 2003, 2008; Schmidt Von Braun and Schleiff, 2008a,b; Kadota et al., 2009), to remodel the actin cytoskeleton and drive chloroplast movement upon light perception by PHOT1 and PHOT2 are central questions that should be addressed in future studies. As already stated in the previous section, class II formins represent additional potential linkers between chloroplasts and actin bundles (Zhang et al., 2011a).

## Vacuolar H^+^-ATPases B subunits

Vacuolar H^+^-ATPases (V-ATPases) are evolutionary-conserved multisubunit complexes that consist in a cytosolic ATP-hydrolyzing V_1_ subcomplex and a membrane-associated proton-translocating V_0_ subcomplex (Nishi and Forgac, 2002; Nelson, 2003; Ma et al., 2011). They mediate ATP-dependent transport of protons across plasma and intracellular membranes and thereby contribute to (1) the acidification of the lumen of various organelles such as vacuoles, secretory vesicles, endosomes, Golgi apparatus, and lysosomes and (2) the production of the energy required for various coupled transport processes. Accordingly, V-ATPases are involved in a wide range of critical processes including membrane trafficking and fusion, and cell expansion (Schumacher et al., 1999; Padmanaban et al., 2004; Dettmer et al., 2006; Brux et al., 2008). In mammals and yeast, both B and C subunits of the V_1_ subcomplex were previously reported to directly bind to F-actin with high affinity (Lee et al., 1999; Holliday et al., 2000; Vitavska et al., 2003, 2005; Chen et al., 2004; Zuo et al., 2008).

Functional studies support that the actin binding activity of V-ATPase B and C subunits is not involved in the regulation of V-ATPase assembly or activity. However, under stress conditions, it provides a significant survival advantage in yeast, supporting that it is biologically relevant (Xu and Forgac, 2001; Zuo et al., 2008). In addition, several studies have highlighted that the targeting of V-ATPases to specific sites relies on their interaction with the actin cytoskeleton (Lee et al., 1999; Adams et al., 2006; Zuo et al., 2006). Carnell et al. (2011) recently suggested a novel and elegant model in which nucleation-promoting factor WASH-dependent actin polymerization on mature lysosomes from *Dictyostelium* would sort V-ATPases to recycling vesicles, leading to subsequent lysosome neutralization and exocytosis. In the absence of WASH, no polymerization would occur and V-ATPases would remain on the lysosome, which in turn would remain acidic and unable to exocytose. Such a model assumes that the actin-binding activity of V-ATPases functions as tags for actin-mediated sorting.

A similar mechanism in plant cells is plausible since homologs of V-ATPases (Zimniak et al., 1988; Krebs et al., 2010), ARP2/3 complex and associated nucleation-promoting factors have been identified (Deeks and Hussey, 2005; Szymanski, 2005). In addition, the three *Arabidopsis* V-ATPase B subunits (*At*AVB1, *At*VAB2, and *At*VAB3) were recently shown to display direct actin binding and bundling activities *in vitro* (Ma et al., 2012). Therefore, the multiple actin-binding sites responsible for the *in vitro* actin bundling activity of *At*AVB1-3 may confer these proteins an increased affinity for actin filaments/bundles and trigger their clustering and/or recycling upon actin polymerization. Such a scenario of an actin-mediated sorting mechanism in plants remains however highly hypothetical. As an alternative, plant V-ATPases might serve as more passive points for anchoring organelles to actin bundles. A last possibility is that *At*AVB1-3 function in a complex dissociated form in the cytoplasm, and therefore contribute to increase actin-bundling upon V-ATPase complex dissociation. Obviously, much work is required to examine each of these possibilities. Nevertheless, V-ATPases emerge as potential additional links between the actin cytoskeleton and membrane trafficking.

## SCAB1

Stomatal movement is driven by modifications in turgor pressure of the guard cells. Stomata open when the guard cell volume increases, and they close when the guard cell volume decreases. It is well established that stomatal closure and opening involves reorganization of the actin cytoskeleton at the cell cortex and that such reorganization plays a key role in stomatal movement (e.g., Kim et al., 1995; Eun and Lee, 1997; Liu and Luan, 1998; Hwang and Lee, 2001; Lemichez et al., 2001; Macrobbie and Kurup, 2007; Choi et al., 2008; Gao et al., 2008). Recently, Higaki et al. (2010b) developed a novel quantitative image analysis method allowing a more detailed and reliable characterization of the changes in actin configurations during the diurnal cycles of *Arabidopsis* guard cells. Data confirmed previous observations that actin filaments adopt a well-organized and radial orientation in open stomata and a more longitudinal orientation in closed stomata. They also provide clear evidence that actin-bundling transiently increases during stomatal opening, and drastically reduces once this process is completed. Interestingly, the abnormally thick and long-lasting actin bundles induced by the expression of a mouse talin-derived actin reporter compromised stomatal opening. This is in good agreement with previous pharmacological and genetic studies indicating that changes in actin dynamics control stomatal movement (Kim et al., 1995; Liu and Luan, 1998; Dong et al., 2001; Lemichez et al., 2001; Macrobbie and Kurup, 2007).

Higaki et al. (2010a,b) suggest that unbundling of actin bundles (rather than their complete depolymerization; Liu and Luan, 1998) stimulates membrane trafficking and increases the number of activated potassium channels in the plasma membrane, which in turn promotes an increase of turgor pressure. In this context, a novel plant-specific actin-bundling protein, termed STOMATAL CLOSURE-RELATED ACTIN BINDING PROTEIN 1 (SCAB1), was isolated from a genetic screen aimed at identifying *Arabidopsis* mutants defective in stomatal movement (Zhao et al., 2011). *In vitro* biochemical data revealed that SCAB1 is a simple actin bundling protein unable to promote actin nucleation or capping. Depletion of SCAB1 reduces actin filament stability, delays the switch from a radial to a longitudinal actin filament configuration in guard cells during stomatal closure, and reduces stomatal closure sensitivity to abscisic acid, H_2_O_2_, and CaCl_2_. In contrast, the overexpression of *SCAB1* increases actin filament stability and promotes excessive bundling. Both *SCAB1* knockout and overexpressing lines exhibit a retardation of stomatal closure, suggesting that proper levels of SCAB1 and actin bundling are required for normal stomatal movements.

Structural and domain analyses indicate that SCAB1 functions as a single actin-binding domain protein that dimerizes through its central coiled coils to achieve the bivalent organization required for actin filament crosslinking (Zhang et al., 2012). Contrary to some other ABPs, SCAB1 activities are insensitive to pH and Ca^2+^ *in vitro* (Zhao et al., 2011). Nevertheless, the SCAB1 C-terminal pleckstrin homology domain was shown to weakly bind to inositol phosphates, suggesting a possible SCAB1 regulation by phosphoinositides at the cell membrane (Zhang et al., 2012). In addition to its potential impact on potassium channel density at the membrane of guard cells, actin bundling might also play a structural role in the control of the vacuolar shape and volume. Indeed, the radial actin filament configuration in open stomata allows the vacuole to occupy a maximal volume. In contrast, the long and heavy bundles spanning along the longitudinal axis of the guard cells of closed stomata might contribute to reduce the vacuole volume. Although the exact roles of actin bundles and the newly discovered actin bundling protein SCAB1 in stomatal movement remain to be established, there is accumulating evidence that they are central players.

## Villins

Plant villins define a class of multifunctional ABPs which can combine several actin regulatory activities, including actin filament severing, barbed-end capping, and bundling activities. The *Arabidopsis* genome contains five villin genes (*AtVLN1-5*), each of which being highly expressed in a wide range of tissues (Klahre et al., 2000; Huang et al., 2005). Whereas atypical AtVLN1 was reported to function as a simple and calcium-insensitive bundling protein (Huang et al., 2005; Khurana et al., 2010), recent biochemical work supports that the rest of the family, including AtVLN2-5, retains the full set of typical villin activities and is calcium-responsive (Khurana et al., 2010; Zhang et al., 2010, 2011b; Bao et al., 2012; van der Honing et al., 2012). The analysis of AtVLN4 and AtVLN5 loss-of-function mutants (Zhang et al., 2010, 2011b) confirmed the predicted roles of villins in the formation and/or stabilization of the long actin bundles running along the shank of pollen tubes and root hairs (e.g., Yokota et al., 1998, 2003; Tominaga et al., 2000; Ketelaar et al., 2002). These studies also further validated the primary role of such actin bundles in the intracellular transport of organelles and vesicles in tip-growing cells (e.g., Miller et al., 1999; Sheahan et al., 2004; Lovy-Wheeler et al., 2005; Ye et al., 2009). Interestingly, the fact that beside their bundling activity, AtVLN4 and AtVLN5 possess calcium-dependent actin severing and capping activities suggests that they also actively contribute to assembling and disassembling the typical short actin bundle-based structures observed in the subapical region of pollen tubes and root hairs (Zhang et al., 2010, 2011b). As these structures remain at a constant distance from the growing cell tip, they inevitably undergo continuous cycles of disassembly/reassembly, a process which is thought to be primarily regulated by changes in the concentration of ions including [Ca^+^] and [H^+^], and reactive oxygen species (Holdaway-Clarke and Hepler, 2003; Knight, 2007; Cheung and Wu, 2008).

The analysis of *vln2vln3* double T-DNA insertion mutants supports that AtVLN2 and AtVLN3 together play a major role in the generation of thick actin bundles in tissues other than pollen and root hairs, and that such bundles are involved in the regulation of directional organ growth (van der Honing et al., 2012). Indeed, unlike single *vln2* and *vln3* mutants, double mutants exhibit much thinner actin bundles as compared to wild type plants, and develop twisted leaves, stems, siliques, and roots. Only full-length AtVLN3, but not a truncated version lacking the headpiece region which is required for actin bundling *in vitro*, could rescue both actin and developmental phenotypes of *vln2vln3* double mutants, supporting that villin-induced thick bundles are required for proper regulation of coordinated cell expansion. It is noteworthy that cell shape and size and plant growth rates are similar in control and double mutant plants, indicating that cell expansion itself is unaffected. Surprisingly, another recent study, which also focused on *vln2vln3* double T-DNA insertion mutants (Bao et al., 2012), reported a morphological phenotype differing from the one described by van der Honing et al. (2012). In this study, the inflorescence stem of *vln2vln3* seedlings developed a pendent phenotype which was correlated to defects in sclerenchyma development (Bao et al., 2012). Although petioles were modestly twisted, this malformation was obviously milder than the prominent twisted phenotype exhibited by various organs of van der Honing's double mutants. Both the pendent and faint twisted phenotypes of Bao's double mutants could be rescued by the expression of either VLN2 or VLN3. In addition, quantitative analyses indicate that xylem fiber cells of double mutant inflorescence stems contain abnormally fine actin bundles, supporting van der Honing's conclusions that VLN2 and VLN3 work as effective and functionally redundant actin-bundling proteins *in vitro*. The morphological differences between the Bao and van der Honing phenotypes remain intriguing and might reflect the presence of truncated forms of VLN3 (the same *vln2* mutant is used as a parental line in both studies) and/or the use of dissimilar plant growth conditions.

## LIM proteins

Plant LIM proteins or LIMs (the acronym LIM derived from the first letter of the three first identified LIM domain-containing proteins, namely LIN-11, ISl1, and MEC-3; Way and Chalfie, 1988; Freyd et al., 1990; Karlsson et al., 1990) define a ubiquitous family of actin-bundling proteins. Since our first report on tobacco NtWLIM1 describing the promotion of actin-bundle formation both *in vitro* and in live cells (Thomas et al., 2006, 2007), several additional LIMs, including the six *Arabidopsis* AtLIMs (Papuga et al., 2010; Ye and Xu, 2012), lilium LlLIM1 (Wang et al., 2008), and tobacco NtWLIM2 (Moes et al., 2012), were biochemically characterized and recognized as actin-bundling proteins. Contrary to formins and villins, no other actin-regulatory activity has been attributed to any plant LIM protein so far, supporting that they function as simple actin bundlers. In Papuga et al. (2010), we showed that the actin bundling activity of the three pollen-enriched *Arabidopsis* LIMs (AtPLIM2a–c) is regulated by pH and calcium (AtPLIM2c), whereas that of the three widely-expressed LIMs (AtWLIM1, AtWLIM2a, and AtWLIM2b) is not. These data are particularly relevant considering the central roles previously proposed for pH and calcium gradients/oscillations in the regulation of ABPs activities and cytoskeletal organization during pollen tube elongation (Staiger et al., 2010). Using an *Arabidopsis* cell suspension-based system, we could demonstrate that the interaction of PLIMs with the actin cytoskeleton can be specifically and reversibly inhibited by a controlled increase of the intracellular pH (Papuga et al., 2010). Overexpression of LlLIM1 modifies the actin cytoskeleton architecture in growing pollen tubes of lily, disturbs endomembrane trafficking, including the Golgi apparatus and endo/exocytic vesicles, and impairs normal targeting of signaling molecules, including phosphatidylinositol-4,5-bisphosphate, phospholipase C, and diacyl glycerol (Wang et al., 2008). As an additional proof of the biological functions of LIMs in pollen, the partial co-suppression of the three AtPLIMs by an RNAi approach was recently shown to provoke important defects in pollen development and tube growth (Ye and Xu, 2012).

Beside their cytosplasmic functions, plant LIMs were repeatedly reported to enter the nucleus, although their roles in this compartment have been comparatively less studied (Mundel et al., 2000; Kawaoka and Ebinuma, 2001; Briere et al., 2003; Thomas et al., 2006; Papuga et al., 2010). One of the first hints of the nuclear roles of plant LIMs was the identification of tobacco NtWLIM1 as a *trans* factor binding to a PAL-box motif of the horseradish *C2 peroxidase* (*prxC2*) gene whose product is involved in phenylpropanoid biosynthesis (Kawaoka et al., 1992, 2000). Supporting the biological relevance of this finding, transgenic tobacco plants with an antisense *NtWLIM1* exhibited abnormally low levels of transcripts of several key phenylpropanoid pathway genes as well as a 27% reduction in lignin content (Kawaoka et al., 2000; Kaothien et al., 2002). We recently evaluated the nuclear functions of the tobacco NtWLIM2 and found that NtWLIM2 can specifically and directly bind to the conserved octamer *cis*-element of the histone *AtH4A748* promoter and activate the corresponding promoter in live cell reporter-based experiments (Moes et al., 2012). Similar activities were also shown for the *Arabidopsis* homolog of NtWLIM2, namely AtWLIM2a, whereas the more distant NtWLIM1 and AtWLIM1 proteins were unable to bind to and to activate the *AtH4A748* promoter, suggesting a specialization of LIM protein subfamilies in their nuclear targets.

Like all the other plant LIMs previously characterized, NtWLIM2 decorates the actin cytoskeleton in live cells, and binds to and bundles actin filaments *in vitro* (Moes et al., 2012). Interestingly, we observed that the NtWLIM2 nuclear fraction readily increases after cell treatment with the F-actin disrupting drug latrunculin B, suggesting that the compartmentalization of NtWLIM2 is modulated by the cytoskeletal status of the cell. It is noteworthy that the mammalian counterparts of plant LIMs, namely the cysteine-rich proteins (CRP1-3) were also reported to shuttle between the cytoplasm and the nucleus where they function as co-activators of genes involved in muscle differentiation (Arber et al., 1994; Arber and Caroni, 1996; Kong et al., 1997; Chang et al., 2003, 2007). In addition, some data support that CRP3 translocates to the nucleus in response to mechanical cues (Boateng et al., 2007, 2009) and that both CRP2 and CRP3 are involved in the stretch response and the regulation of the cell contractile force through their interaction with actin stress fibers (Knoll et al., 2002; Kim-Kaneyama et al., 2005). It is therefore tempting to propose that plant LIMs function as sensors able to perceive mechanical signals and to regulate in turn the mechanical properties of the cell by regulating gene expression (Kawaoka et al., 2000; Kaothien et al., 2002) and remodeling the actin cytoskeleton. In addition, recent expression analyses have highlighted that a subset of poplar LIMs is up-regulated in tension wood (Arnaud et al., 2012), further indicating a connection between plant LIMs and mechanical stress. Such a hypothesis is currently tested in our lab.

## Fimbrins

Fimbrins (also known as plastins in humans) define an evolutionary-conserved family of actin bundling proteins whose activities, biological functions, and roles in diseases have been extensively analyzed in animals/humans and yeast (e.g., Bretscher, 1981; Samstag and Klemke, 2007; Al Tanoury et al., 2010; Skau et al., 2011; Morley, 2012; Shinomiya, 2012). The *Arabidopsis* genome encodes five fimbrins (AtFIM1-5; Staiger and Hussey, 2004). Although the structural bases underlying the actin binding and crosslinking activities of AtFIM1 were characterized in detail, only few studies have directly addressed the biological functions of plant fimbrins (Kovar et al., 2000, 2001; Klein et al., 2004; Wang et al., 2004). Wu et al. (2010) recently provided evidence that pollen-enriched AtFIM5 is required for the proper organization of the actin cytoskeleton in pollen grains and growing pollen tubes. The loss of AtFIM5 disorganizes the typical longitudinal configuration of actin bundles in the shank of the pollen tube and causes some bundles to invade the extreme tip. Such aberrant cytoskeletal organization in turn alters the pattern and velocity of cytoplasmic streaming. Biochemical data revealed that AtFIM5 is a calcium-insensitive actin bundling factor. Although the mechanism by which the loss-of-function of an actin-bundling protein leads to an increase in actin bundles a the tip of pollen tubes remains obscure, together these data highlight an important role for FIM5 in maintaining the normal actin organization and/or dynamics in pollen tubes.

## SB401

SB401 is a pollen-specific protein from *Solanum berthaultii* (Liu et al., 1997) which was previously reported to bind to and bundle both microtubules and actin filaments and proposed to function as a linker between microtubule and actin cytoskeletons (Huang et al., 2007). In agreement with its higher *in vitro* affinity for microtubules, SB401 was observed to preferentially interact with the microtubule cytoskeleton in immunolabeled pollen tubes. However, recent *in vitro* biochemical analyses support that phosphorylation of SB401 by casein kinase II specifically inhibits SB401 microtubule regulatory activities, suggesting that phosphorylation can switch the protein toward its actin regulatory function(s) (Liu et al., 2009). Future work should validate SB401 cytoskeleton regulatory activities in a live cell context and provide an insight into its biological function(s) in potato pollen tubes.

## AtADF9

Members of the ADF/cofilin family are well-established ABPs able to bind both actin monomers and filaments and whose main function is to increase actin dynamics (Staiger and Blanchoin, 2006; Ono, 2007; Van Troys et al., 2008b; Bernstein and Bamburg, 2010). Whereas vertebrates typically possess three ADFs/cofilins, plant ADF families are particularly large. Indeed, *Arabidopsis* expresses 11 functional ADFs (AtADF1-11) which can be divided into 5 subclasses according to their tissular expression and phylogeny (Ruzicka et al., 2007). Recently, time-lapse TIRFM analyses provided direct evidence that subclass I AtADF1 and AtADF4 sever actin filaments *in vitro* (Khurana et al., 2010; Henty et al., 2011), an activity displayed by most animal, protozoa, and yeast ADFs/cofilins (e.g., Andrianantoandro and Pollard, 2006; Chan et al., 2009). In agreement with these data and the role predicted for actin severing in the stochastic dynamics of plant actin filaments (Michelot et al., 2007; Blanchoin et al., 2010; Staiger et al., 2010), Henty et al. (2011) established that *Arabidopsis adf4* knockout mutants exhibit a 2.5-fold reduced severing frequency as well as other characteristics of reduced actin dynamics in the cortical region of hypocotyl epidermal cells.

We recently compared the biochemical activities of *Arabidopsis* ADFs from different subclasses (unpublished data). We found that, contrary to other ADFs, subclass III AtADF9 is unable to enhance actin depolymerization *in vitro* (Tholl et al., 2011). Instead, AtADF9 stabilizes and crosslinks actin filaments into large bundles. By transiently expressing GFP-tagged and untagged AtADF9 recombinant proteins in tobacco BY2 cells, we confirmed the actin-bundling activity of AtADF9 in a live cell context. Indeed, contrary to AtADF1 which induced many breaks in the actin cytoskeleton, AtADF9 reduced the density and increased the thickness of actin fibers. Interestingly, similar data were obtained with AtADF5 (unpublished data), the other member of *Arabidopsis* ADF subclass III. Future work should identify the structural features responsible for the unconventional activities of subclass III ADFs, and compare the developmental and actin cytoskeleton phenotypes of *adf5* and *adf9* mutants to those recently reported for the knockout mutant of the conventional ADF AtADF4 (Henty et al., 2011).

Table 1 lists the actin bundling proteins cited in the present article and emphasizes some of their important features.

**Table 1:** **List of the actin bundling promoting proteins cited in this article**.

**Name**	**Remarkable features**	**Reported subcellular locations**	**Identified or suggested biological functions**	**Key references**
**FORMINS**				
AtFH1	Non-processive formin; anchors in the cell wall	Cell membrane	Pollen tube growth, cell expansion	Cheung and Wu, 2004; Michelot et al., 2005, 2006; Martiniere et al., 2011
AtFH3	Lacks *in vitro* actin bundling activity	Nuclear envelope; cell plate	Pollen tube growth polarity	Ye et al., 2009
AtFH4	Bundles both AFs and MTs; AtFH4-GFP co-aligns the ER and MTs	Cell membrane at cell-to-cell contacts; ER membrane	Cell expansion	Deeks et al., 2005, 2010
AtFH5	*In vitro* actin bundling activity not reported so far	Growing cell plate; cell membrane in the pollen tube tip	Cell cytokinesis; pollen tube growth	Ingouff et al., 2005; Cheung et al., 2010
AtFH8	AtFH8(FH1FH2) induces stellar structures *in vitro*	Cell membrane at cell-to-cell contacts; nuclear envelope	Primary root growth; lateral root initiation; cell expansion and division	Deeks et al., 2005; Yi et al., 2005; Xue et al., 2011
AtFH14	Bundles both AFs and MTs; crosslinks AFs and MTs together	Preprophase band; phragmoplast	Cell division	Li et al., 2010
OsFH5	Bundles both AFs and MTs	Chloroplast surface	Cell expansion	Yang et al., 2011; Zhang et al., 2011a
AtTHRUMIN1	Light-dependent actin bundling activity	Cell membrane	Chloroplast movement	Whippo et al., 2011
AtAVB1-3	Part of the V-ATPase multimeric complex	Endomembrane system	–	Ma et al., 2012
AtSCAB1	Dimerizes; likely regulated by phosphoinositides	Cytoplasm	Stomatal movement	Zhao et al., 2011; Zhang et al., 2012
**VILLINS**				
LlP-135-ABP and LlP-115-ABP	Ca^2+^ sensitive; bundle AFs with uniform polarity	Cytoplasm	Direction of cytoplasmic streaming in pollen tubes and root hair cells	Yokota et al., 1998, 2000, 2003, 2005; Yokota and Shimmen, 1999; Tominaga et al., 2000
AtVLN1	Ca^2+^ insensitive; lacks severing and capping activities	–	–	Huang et al., 2005; Khurana et al., 2010
AtVLN2	Ca^2+^ sensitive; has severing and capping activities	Cytoplasm	Directional organ growth; Sclerenchyma development	Bao et al., 2012; van der Honing et al., 2012
AtVLN3	Ca^2+^ sensitive; has severing and capping activities; can sever AtVLN1-induced bundles *in vitro*	Cytoplasm	Directional organ growth; Sclerenchyma development	Khurana et al., 2010; Bao et al., 2012; van der Honing et al., 2012
AtVLN4	Ca^2+^ sensitive; has severing and capping activities	Cytoplasm	Root hair growth and cytoplasmic streaming	Zhang et al., 2011b
AtVLN5	Ca^2+^ sensitive; has severing and capping activities	Cytoplasm	Pollen tube growth	Zhang et al., 2010
**LIM PROTEINS**				
NtWLIM1	Interacts directly with DNA	Cytoplasm; nucleus	Gene expression (lignin biosynthesis)	Kawaoka et al., 2000; Kaothien et al., 2002; Thomas et al., 2006, 2007
NtWLIM2	Interacts directly with DNA; dimerizes	Cytoplasm; nucleus	Gene expression (Histones)	Moes et al., 2012
AtWLIM1, 2a and b	Ca^2+^ and pH insensitive	Cytoplasm; nucleus	–	Papuga et al., 2010
AtPLIM2a and b	Only pH sensitive	Cytoplasm; nucleus	Pollen tube growth	Papuga et al., 2010; Ye and Xu, 2012
AtPLIM2c	Ca^2+^ and pH sensitive	Cytoplasm; nucleus	Pollen tube growth	Papuga et al., 2010; Ye and Xu, 2012
LlLIM1	Ca^2+^ and pH sensitive	Cytoplasm; nucleus	Pollen tube growth	Wang et al., 2008
**FIMBRINS**				
AtFIM1	Ca^2+^ insensitive	Cytoplasm	Cytoplasmic streaming	Kovar et al., 2000, 2001
AtFIM5	Ca^2+^ insensitive	Cytoplasm	Pollen tube germination and growth	Wu et al., 2010
**OTHER ABP FAMILIES**				
Sb401	Bundles both AFs and MTs; activity possibly switched toward actin bundling by phosphorylation; genus-specific protein	Cytoplasm; cell cortex	–	Huang et al., 2007; Liu et al., 2009
AtADF9	Expression induced by hormones; lacks conventional ADF AF severing activity	Cytoplasm; nucleus	Gene expression (repression of flowering); development	Burgos-Rivera et al., 2008; Tholl et al., 2011

In the column “Reported subcellular locations,” the term “cytoplasm” means no association with any specific organelle. Note that, in some cases, the “Identified or suggested biological functions” is not directly related to the actin bundling activity of the protein, e.g., nuclear functions. AFs, actin filaments; ER, endoplasmic reticulum; MT, microtubules.

## Conclusions

The growing number and diversity of actin-bundling proteins identified in plants indicate that, like animals, plants elaborate various types of actin-bundles with specific structural features and distinct functions (Table 1). This implies that the functions of actin bundles extend beyond the traditional definition of stable tracks for long distance intracellular transport. The characterization of novel types of actin-bundling proteins points out potential functions for actin bundles in stomatal movement, ion channel trafficking and/or activities, and chloroplast movement. In addition, actin bundles most likely play an important role in the regulation of hormone carriers cycling between plasma membrane and intracellular compartments (Nick, 2010). The specific actin bundling proteins involved in this process as well as their mode of regulation however remain to be identified.

Precise actin filament dynamics and organization near the plant cell cortex have been resolved only recently, and the elucidation of the roles of actin filaments and bundles at this location will keep researchers busy during the years to come. Future work should establish why epidermal plant cells keep their cortical actin network so dynamic and whether the other cell types do the same. A number of actin-bundling proteins reviewed in this article support the existence of a physical linkage between actin bundles and membranes. Among those, formins emerge as key multifunctional ABPs able to initiate polymerization and bundling of filaments from diverse types of subcellular locations including the cell membrane. Noticeably, the recent work by Martiniere et al. (2011) provides compelling evidence that the *Arabidopsis* formin AtAFH1 is anchored by its predicted extracellular domain within the cell wall and bridges the latter to the actin cytoskeleton. A next important step consists in identifying the external signals that target the extracellular domain of class I formins and in characterizing how such signals modulate the intracellular activities of formins. In addition, one can expect that, following the pioneering work on AtADF4 by Henty et al. (2011), the exact contribution of formins to actin nucleation and bundling near the cell cortex, and more generally to the actin stochastic dynamics, will be soon characterized. During the reviewing process of the present article, a publication by Deeks et al. (2012) reporting the identification of a novel and plant-specific superfamily of ABPs termed Networked (NET) was released. Localization analyses strongly suggest that the *Arabidopsis* NET proteins function as linkers between the actin cytoskeleton and diverse types of membranes, including specific subdomains of the plasma membrane, the tonoplast and the nuclear membrane. There is no doubt that such an exciting discovery will boost the field and contribute to a better understanding of how AFs and actin bundles are coupled to membranes in plant cells.

### Conflict of interest statement

The author declares that the research was conducted in the absence of any commercial or financial relationships that could be construed as a potential conflict of interest.

## References

[B1] Adams D. S. Robinson K. R. Fukumoto T. Yuan S. Albertson R. C. Yelick P. Kuo L. McSweeney M. Levin M. (2006). Early, H+-V-ATPase-dependent proton flux is necessary for consistent left-right patterning of non-mammalian vertebrates. Development 133, 1657–1671 10.1242/dev.02341 16554361 PMC3136117

[B2] Alexandersson E. Saalbach G. Larsson C. Kjellbom P. (2004). Arabidopsis plasma membrane proteomics identifies components of transport, signal transduction and membrane trafficking. Plant Cell Physiol. 45, 1543–1556 10.1093/pcp/pch209 15574830

[B3] Al Tanoury Z. Schaffner-Reckinger E. Halavatyi A. Hoffmann C. Moes M. Hadzic E. Catillon M. Yatskou M. Friederich E. (2010). Quantitative kinetic study of the actin-bundling protein L-plastin and of its impact on actin turn-over. PLoS ONE 5:e9210 10.1371/journal.pone.0009210 20169155 PMC2821400

[B4] Andrianantoandro E. Pollard T. D. (2006). Mechanism of actin filament turnover by severing and nucleation at different concentrations of ADF/cofilin. Mol. Cell 24, 13–23 10.1016/j.molcel.2006.08.006 17018289

[B5] Arber S. Caroni P. (1996). Specificity of single LIM motifs in targeting and LIM/LIM interactions *in situ*. Genes Dev. 10, 289–300 10.1101/gad.10.3.289 8595880

[B6] Arber S. Halder G. Caroni P. (1994). Muscle LIM protein, a novel essential regulator of myogenesis, promotes myogenic differentiation. Cell 79, 221–231 10.1016/0092-8674(94)90192-9 7954791

[B7] Arnaud D. Dejardin A. Leple J. C. Lesage-Descauses M. C. Boizot N. Villar M. Benedetti H. Pilate G. (2012). Expression analysis of LIM gene family in poplar, toward an updated phylogenetic classification. BMC Res. Notes 5, 102 10.1186/1756-0500-5-102 22339987 PMC3392731

[B8] Avisar D. Prokhnevsky A. I. Makarova K. S. Koonin E. V. Dolja V. V. (2008). Myosin XI-K is required for rapid trafficking of Golgi stacks, peroxisomes, and mitochondria in leaf cells of *Nicotiana benthamiana*. Plant Physiol. 146, 1098–1108 10.1104/pp.107.113647 18178670 PMC2259067

[B9] Baluska F. Jasik J. Edelmann H. G. Salajova T. Volkmann D. (2001). Latrunculin B-induced plant dwarfism: plant cell elongation is F-actin-dependent. Dev. Biol. 231, 113–124 10.1006/dbio.2000.0115 11180956

[B10] Baluska F. Samaj J. Wojtaszek P. Volkmann D. Menzel D. (2003). Cytoskeleton-plasma membrane-cell wall continuum in plants. Emerging links revisited. Plant Physiol. 133, 482–491 10.1104/pp.103.027250 14555777 PMC523875

[B11] Banno H. Chua N. H. (2000). Characterization of the arabidopsis formin-like protein AFH1 and its interacting protein. Plant Cell Physiol. 41, 617–626 10929945 10.1093/pcp/41.5.617

[B12] Bao C. Wang J. Zhang R. Zhang B. Zhang H. Zhou Y. Huang S. (2012). Arabidopsis VILLIN2 and VILLIN3 act redundantly in sclerenchyma development via bundling of actin filaments. Plant J. [Epub ahead of print]. 10.1111/j.1365-313X.2012.05044.x 22563899

[B13] Bernstein B. W. Bamburg J. R. (2010). ADF/cofilin: a functional node in cell biology. Trends Cell Biol. 20, 187–195 10.1016/j.tcb.2010.01.001 20133134 PMC2849908

[B14] Blanchoin L. Boujemaa-Paterski R. Henty J. L. Khurana P. Staiger C. J. (2010). Actin dynamics in plant cells: a team effort from multiple proteins orchestrates this very fast-paced game. Curr. Opin. Plant Biol. 13, 714–723 10.1016/j.pbi.2010.09.013 20970372

[B15] Blanchoin L. Staiger C. J. (2008). Plant formins: diverse isoforms and unique molecular mechanism. Biochim. Biophys. Acta. 1803, 201–206 10.1016/j.bbamcr.2008.09.015 18977251

[B16] Boateng S. Y. Belin R. J. Geenen D. L. Margulies K. B. Martin J. L. Hoshijima M. De Tombe P. P. Russell B. (2007). Cardiac dysfunction and heart failure are associated with abnormalities in the subcellular distribution and amounts of oligomeric muscle LIM protein. Am. J. Physiol. Heart Circ. Physiol. 292, H259–H269 10.1152/ajpheart.00766.2006 16963613

[B17] Boateng S. Y. Senyo S. E. Qi L. Goldspink P. H. Russell B. (2009). Myocyte remodeling in response to hypertrophic stimuli requires nucleocytoplasmic shuttling of muscle LIM protein. J. Mol. Cell. Cardiol. 47, 426–435 10.1016/j.yjmcc.2009.04.006 19376126 PMC2739242

[B18] Bretscher A. (1981). Fimbrin is a cytoskeletal protein that crosslinks F-actin *in vitro*. Proc. Natl. Acad. Sci. U.S.A. 78, 6849–6853 6947259 10.1073/pnas.78.11.6849 PMC349149

[B19] Briere C. Bordel A. C. Barthou H. Jauneau A. Steinmetz A. Alibert G. Petitprez M. (2003). Is the LIM-domain protein HaWLIM1 associated with cortical microtubules in sunflower protoplasts? Plant Cell Physiol. 44, 1055–1063 10.1093/pcp/pcg126 14581630

[B20] Brux A. Liu T. Y. Krebs M. Stierhof Y. D. Lohmann J. U. Miersch O. Wasternack C. Schumacher K. (2008). Reduced V-ATPase activity in the trans-Golgi network causes oxylipin-dependent hypocotyl growth Inhibition in Arabidopsis. Plant Cell 20, 1088–1100 10.1105/tpc.108.058362 18441211 PMC2390726

[B21] Burgos-Rivera B. Ruzicka D. R. Deal R. B. McKinney E. C. King-Reid L. Meagher R. B. (2008). ACTIN DEPOLYMERIZING FACTOR9 controls development and gene expression in Arabidopsis. Plant Mol. Biol. 68, 619–632 10.1007/s11103-008-9398-1 18830798 PMC2811079

[B22] Carnell M. Zech T. Calaminus S. D. Ura S. Hagedorn M. Johnston S. A. May R. C. Soldati T. Machesky L. M. Insall R. H. (2011). Actin polymerization driven by WASH causes V-ATPase retrieval and vesicle neutralization before exocytosis. J. Cell Biol. 193, 831–839 10.1083/jcb.201009119 21606208 PMC3105540

[B23] Chan C. Beltzner C. C. Pollard T. D. (2009). Cofilin dissociates Arp2/3 complex and branches from actin filaments. Curr. Biol. 19, 537–545 10.1016/j.cub.2009.02.060 19362000 PMC3711486

[B24] Chang D. F. Belaguli N. S. Chang J. Schwartz R. J. (2007). LIM-only protein, CRP2, switched on smooth muscle gene activity in adult cardiac myocytes. Proc. Natl. Acad. Sci. U.S.A. 104, 157–162 10.1073/pnas.0605635103 17185421 PMC1765427

[B25] Chang D. F. Belaguli N. S. Iyer D. Roberts W. B. Wu S. P. Dong X. R. Marx J. G. Moore M. S. Beckerle M. C. Majesky M. W. Schwartz R. J. (2003). Cysteine-rich LIM-only proteins CRP1 and CRP2 are potent smooth muscle differentiation cofactors. Dev. Cell 4, 107–118 10.1016/S1534-5807(02)00396-9 12530967

[B26] Chen S. H. Bubb M. R. Yarmola E. G. Zuo J. Jiang J. Lee B. S. Lu M. Gluck S. L. Hurst I. R. Holliday L. S. (2004). Vacuolar H+-ATPase binding to microfilaments: regulation in response to phosphatidylinositol 3-kinase activity and detailed characterization of the actin-binding site in subunit B. J. Biol. Chem. 279, 7988–7998 10.1074/jbc.M305351200 14662773

[B27] Cheung A. Y. Niroomand S. Zou Y. Wu H. M. (2010). A transmembrane formin nucleates subapical actin assembly and controls tip-focused growth in pollen tubes. Proc. Natl. Acad. Sci. U.S.A. 107, 16390–16395 10.1073/pnas.1008527107 20805480 PMC2941322

[B28] Cheung A. Y. Wu H. M. (2004). Overexpression of an Arabidopsis formin stimulates supernumerary actin cable formation from pollen tube cell membrane. Plant Cell 16, 257–269 10.1105/tpc.016550 14671023 PMC301409

[B29] Cheung A. Y. Wu H. M. (2008). Structural and signaling networks for the polar cell growth machinery in pollen tubes. Annu. Rev. Plant Biol. 59, 547–572 10.1146/annurev.arplant.59.032607.092921 18444907

[B30] Choi Y. Lee Y. Jeon B. W. Staiger C. J. (2008). Phosphatidylinositol 3- and 4-phosphate modulate actin filament reorganization in guard cells of day flower. Plant Cell Environ. 31, 366–377 10.1111/j.1365-3040.2007.01769.x 18088331

[B31] Chuong S. D. Franceschi V. R. Edwards G. E. (2006). The cytoskeleton maintains organelle partitioning required for single-cell C4 photosynthesis in Chenopodiaceae species. Plant Cell 18, 2207–2223 10.1105/tpc.105.036186 16905659 PMC1560926

[B32] Ciobanasu C. Faivre B. Le Clainche C. (2012). Actin dynamics associated with focal adhesions. Int. J. Cell Biol. 2012, 941292 10.1155/2012/941292 22505938 PMC3312244

[B33] Collings D. (2008). Crossed-wires: interactions and cross-talk between the microtubule and microfilament networks in plants, in Plant Microtubules, Plant Cell Monographs, ed N. Peter (Berlin: Springer), 47–79

[B34] Crowell E. F. Bischoff V. Desprez T. Rolland A. Stierhof Y. D. Schumacher K. Gonneau M. Hofte H. Vernhettes S. (2009). Pausing of Golgi bodies on microtubules regulates secretion of cellulose synthase complexes in Arabidopsis. Plant Cell 21, 1141–1154 10.1105/tpc.108.065334 19376932 PMC2685615

[B35] Cvrckova F. (2000). Are plant formins integral membrane proteins? Genome Biol. 1, RESEARCH001 11104517 10.1186/gb-2000-1-1-research001 PMC31918

[B36] Day B. Henty J. L. Porter K. J. Staiger C. J. (2011). The pathogen-actin connection: a platform for defense signaling in plants. Annu. Rev. Phytopathol. 49, 483–506 10.1146/annurev-phyto-072910-095426 21495845

[B36a] Deeks M. J. Calcutt J. R. Ingle E. K. Hawkins T. J. Chapman S. Richardson A. C. Mentlak D. A. Dixon M. R. Cartwright F. Smertenko A. P. Oparka K. Hussey P. J. (2012). A Superfamily of Actin-Binding Proteins at the Actin-Membrane Nexus of Higher Plants. Curr. Biol. [Epub ahead of print]. 10.1016/j.cub.2012.06.041 22840520

[B37] Deeks M. J. Cvrckova F. Machesky L. M. Mikitova V. Ketelaar T. Zarsky V. Davies B. Hussey P. J. (2005). Arabidopsis group Ie formins localize to specific cell membrane domains, interact with actin-binding proteins and cause defects in cell expansion upon aberrant expression. New Phytol. 168, 529–540 10.1111/j.1469-8137.2005.01582.x 16313636

[B38] Deeks M. J. Fendrych M. Smertenko A. Bell K. S. Oparka K. Cvrckova F. Zarsky V. Hussey P. J. (2010). The plant formin AtFH4 interacts with both actin and microtubules, and contains a newly identified microtubule-binding domain. J. Cell Sci. 123, 1209–1215 10.1242/jcs.065557 20332108

[B39] Deeks M. J. Hussey P. J. (2005). Arp2/3 and SCAR: plants move to the fore. Nat. Rev. Mol. Cell Biol. 6, 954–964 10.1038/nrm1765 16341081

[B40] Deeks M. J. Hussey P. J. Davies B. (2002). Formins: intermediates in signal-transduction cascades that affect cytoskeletal reorganization. Trends Plant Sci. 7, 492–498 10.1016/S1360-1385(02)02341-5 12417149

[B41] Dettmer J. Hong-Hermesdorf A. Stierhof Y. D. Schumacher K. (2006). Vacuolar H+-ATPase activity is required for endocytic and secretory trafficking in Arabidopsis. Plant Cell 18, 715–730 10.1105/tpc.105.037978 16461582 PMC1383645

[B42] Dong C. H. Xia G. X. Hong Y. Ramachandran S. Kost B. Chua N. H. (2001). ADF proteins are involved in the control of flowering and regulate F-actin organization, cell expansion, and organ growth in Arabidopsis. Plant Cell 13, 1333–1346 10.1105/TPC.010051 11402164 PMC135580

[B43] Drobak B. K. Franklin-Tong V. E. Staiger C. J. (2004). The role of the actin cytoskeleton in plant cell signaling. New Phytol. 163, 13–30 10.1111/j.1469-8137.2004.01076.x 33873778

[B44] Eun S. O. Lee Y. (1997). Actin filaments of guard cells are reorganized in response to light and abscisic acid. Plant Physiol. 115, 1491–1498 10.1104/pp.115.4.1491 9414559 PMC158614

[B45] Favery B. Chelysheva L. A. Lebris M. Jammes F. Marmagne A. De Almeida-Engler J. Lecomte P. Vaury C. Arkowitz R. A. Abad P. (2004). Arabidopsis formin AtFH6 is a plasma membrane-associated protein upregulated in giant cells induced by parasitic nematodes. Plant Cell 16, 2529–2540 10.1105/tpc.104.024372 15319477 PMC520950

[B45a] Freyd G. Kim S. K. Horvitz H. R. (1990). Novel cysteine-rich motif and homeodomain in the product of the Caenorhabditis elegans cell lineage gene lin-11. Nature 344, 876–879 10.1038/344876a0 1970421

[B46] Fu Y. (2010). The actin cytoskeleton and signaling network during pollen tube tip growth. J. Integr. Plant Biol. 52, 131–137 10.1111/j.1744-7909.2010.00922.x 20377675

[B47] Fu Y. Gu Y. Zheng Z. Wasteneys G. Yang Z. (2005). Arabidopsis interdigitating cell growth requires two antagonistic pathways with opposing action on cell morphogenesis. Cell 120, 687–700 10.1016/j.cell.2004.12.026 15766531

[B48] Gao X. Q. Chen J. Wei P. C. Ren F. Wang X. C. (2008). Array and distribution of actin filaments in guard cells contribute to the determination of stomatal aperture. Plant Cell Rep. 27, 1655–1665 10.1007/s00299-008-0581-2 18612643

[B49] Geiger B. Spatz J. P. Bershadsky A. D. (2009). Environmental sensing through focal adhesions. Nat. Rev. Mol. Cell Biol. 10, 21–33 10.1038/nrm2593 19197329

[B50] Hardham A. R. Jones D. A. Takemoto D. (2007). Cytoskeleton and cell wall function in penetration resistance. Curr. Opin. Plant Biol. 10, 342–348 10.1016/j.pbi.2007.05.001 17627866

[B51] Hardham A. R. Takemoto D. White R. G. (2008). Rapid and dynamic subcellular reorganization following mechanical stimulation of Arabidopsis epidermal cells mimics responses to fungal and oomycete attack. BMC Plant Biol. 8, 63 10.1186/1471-2229-8-63 18513448 PMC2435237

[B52] Henty J. L. Bledsoe S. W. Khurana P. Meagher R. B. Day B. Blanchoin L. Staiger C. J. (2011). Arabidopsis actin depolymerizing factor4 modulates the stochastic dynamic behavior of actin filaments in the cortical array of epidermal cells. Plant Cell 23, 3711–3726 10.1105/tpc.111.090670 22010035 PMC3229145

[B53] Higaki T. Kojo K. H. Hasezawa S. (2010a). Critical role of actin bundling in plant cell morphogenesis. Plant Signal. Behav. 5, 1–5 20118661 10.4161/psb.10947 PMC7080472

[B54] Higaki T. Kutsuna N. Sano T. Kondo N. Hasezawa S. (2010b). Quantification and cluster analysis of actin cytoskeletal structures in plant cells: role of actin bundling in stomatal movement during diurnal cycles in Arabidopsis guard cells. Plant J. 61, 156–165 10.1111/j.1365-313X.2009.04032.x 20092030

[B55] Higaki T. Kurusu T. Hasezawa S. Kuchitsu K. (2011). Dynamic intracellular reorganization of cytoskeletons and the vacuole in defense responses and hypersensitive cell death in plants. J. Plant Res. 124, 315–324 10.1007/s10265-011-0408-z 21409543

[B56] Holdaway-Clarke T. L. Hepler P. K. (2003). Control of pollen tube growth: role of ion gradients and fluxes. New Phytol. 159, 539–563 10.1046/j.1469-8137.2003.00847.x 33873604

[B57] Holliday L. S. Lu M. Lee B. S. Nelson R. D. Solivan S. Zhang L. Gluck S. L. (2000). The amino-terminal domain of the B subunit of vacuolar H+-ATPase contains a filamentous actin binding site. J. Biol. Chem. 275, 32331–32337 10.1074/jbc.M004795200 10915794

[B58] Huang S. Jin L. Du J. Li H. Zhao Q. Ou G. Ao G. Yuan M. (2007). SB401, a pollen-specific protein from *Solanum berthaultii*, binds to and bundles microtubules and F-actin. Plant J. 51, 406–418 10.1111/j.1365-313X.2007.03153.x 17559515

[B59] Huang S. Robinson R. C. Gao L. Y. Matsumoto T. Brunet A. Blanchoin L. Staiger C. J. (2005). Arabidopsis VILLIN1 generates actin filament cables that are resistant to depolymerization. Plant Cell 17, 486–501 10.1105/tpc.104.028555 15659626 PMC548821

[B60] Humphrey T. V. Bonetta D. T. Goring D. R. (2007). Sentinels at the wall: cell wall receptors and sensors. New Phytol. 176, 7–21 10.1111/j.1469-8137.2007.02192.x 17803638

[B61] Hwang J. U. Lee Y. (2001). Abscisic acid-induced actin reorganization in guard cells of dayflower is mediated by cytosolic calcium levels and by protein kinase and protein phosphatase activities. Plant Physiol. 125, 2120–2128 10.1104/pp.125.4.2120 11299391 PMC88867

[B62] Ingouff M. Fitz Gerald J. N. Guerin C. Robert H. Sorensen M. B. Van Damme D. Geelen D. Blanchoin L. Berger F. (2005). Plant formin AtFH5 is an evolutionarily conserved actin nucleator involved in cytokinesis. Nat. Cell Biol. 7, 374–380 10.1038/ncb1238 15765105

[B63] Kadota A. Yamada N. Suetsugu N. Hirose M. Saito C. Shoda K. Ichikawa S. Kagawa T. Nakano A. Wada M. (2009). Short actin-based mechanism for light-directed chloroplast movement in Arabidopsis. Proc. Natl. Acad. Sci. U.S.A. 106, 13106–13111 10.1073/pnas.0906250106 19620714 PMC2722281

[B64] Kaothien P. Kawaoka A. Ebinuma H. Yoshida K. Shinmyo A. (2002). Ntlim1, a PAL-box binding factor, controls promoter activity of the horseradish wound-inducible peroxidase gene. Plant Mol. Biol. 49, 591–599 10.1023/A:1015504515492 12081367

[B64a] Karlsson O. Thor S. Norberg T. Ohlsson H. Edlund T. (1990). Insulin gene enhancer binding protein Isl-1 is a member of a novel class of proteins containing both a homeo- and a Cys-His domain. Nature 344, 879–882 10.1038/344879a0 1691825

[B65] Kasahara M. Kagawa T. Oikawa K. Suetsugu N. Miyao M. Wada M. (2002). Chloroplast avoidance movement reduces photodamage in plants. Nature 420, 829–832 10.1038/nature01213 12490952

[B66] Kawaoka A. Ebinuma H. (2001). Transcriptional control of lignin biosynthesis by tobacco LIM protein. Phytochemistry 57, 1149–1157 10.1016/S0031-9422(01)00054-1 11430987

[B67] Kawaoka A. Kaothien P. Yoshida K. Endo S. Yamada K. Ebinuma H. (2000). Functional analysis of tobacco LIM protein Ntlim1 involved in lignin biosynthesis. Plant J. 22, 289–301 10.1046/j.1365-313x.2000.00737.x 10849346

[B68] Kawaoka A. Sato S. Nakahara K. Matsushima N. Okada N. Sekine M. Shinmyo A. Takano M. (1992). Expression and promoter activity of genes for isozymes of horseradish peroxidase. Plant Cell Physiol. 33, 1143–1150

[B69] Ketelaar T. Faivre-Moskalenko C. Esseling J. J. De Ruijter N. C. Grierson C. S. Dogterom M. Emons A. M. (2002). Positioning of nuclei in Arabidopsis root hairs: an actin-regulated process of tip growth. Plant Cell 14, 2941–2955 12417712 10.1105/tpc.005892 PMC152738

[B70] Khurana P. Henty J. L. Huang S. Staiger A. M. Blanchoin L. Staiger C. J. (2010). Arabidopsis VILLIN1 and VILLIN3 have overlapping and distinct activities in actin bundle formation and turnover. Plant Cell 22, 2727–2748 10.1105/tpc.110.076240 20807878 PMC2947172

[B71] Kim M. Hepler P. K. Eun S. O. Ha K. S. Lee Y. (1995). Actin filaments in mature guard cells are radially distributed and involved in stomatal movement. Plant Physiol. 109, 1077–1084 10.1104/pp.109.3.1077 12228654 PMC161411

[B72] Kim-Kaneyama J. R. Suzuki W. Ichikawa K. Ohki T. Kohno Y. Sata M. Nose K. Shibanuma M. (2005). Uni-axial stretching regulates intracellular localization of Hic-5 expressed in smooth-muscle cells *in vitro*. J. Cell Sci. 118, 937–949 10.1242/jcs.01683 15713747

[B73] Klahre U. Friederich E. Kost B. Louvard D. Chua N. H. (2000). Villin-like actin-binding proteins are expressed ubiquitously in Arabidopsis. Plant Physiol. 122, 35–48 10.1104/pp.122.1.35 10631247 PMC58842

[B74] Klein M. G. Shi W. Ramagopal U. Tseng Y. Wirtz D. Kovar D. R. Staiger C. J. Almo S. C. (2004). Structure of the actin crosslinking core of fimbrin. Structure (Camb.) 12, 999–1013 10.1016/j.str.2004.04.010 15274920

[B75] Knight M. R. (2007). New ideas on root hair growth appear from the flanks. Proc. Natl. Acad. Sci. U.S.A. 104, 20649–20650 10.1073/pnas.0710632105 18093910 PMC2409207

[B76] Knoll R. Hoshijima M. Hoffman H. M. Person V. Lorenzen-Schmidt I. Bang M. L. Hayashi T. Shiga N. Yasukawa H. Schaper W. McKenna W. Yokoyama M. Schork N. J. Omens J. H. McCulloch A. D. Kimura A. Gregorio C. C. Poller W. Schaper J. Schultheiss H. P. Chien K. R. (2002). The cardiac mechanical stretch sensor machinery involves a Z disc complex that is defective in a subset of human dilated cardiomyopathy. Cell 111, 943–955 10.1016/S0092-8674(02)01226-6 12507422

[B77] Kobayashi I. Kobayashi Y. Hardham A. R. (1994). Dynamic reorganization of microtubules and microfilaments in flax cells during the resistance response to flax rust infection. Planta 195, 237–247

[B78] Kong Y. Flick M. J. Kudla A. J. Konieczny S. F. (1997). Muscle LIM protein promotes myogenesis by enhancing the activity of MyoD. Mol. Cell. Biol. 17, 4750–4760 9234731 10.1128/mcb.17.8.4750 PMC232327

[B79] Kovar D. R. Gibbon B. C. McCurdy D. W. Staiger C. J. (2001). Fluorescently-labeled fimbrin decorates a dynamic actin filament network in live plant cells. Planta 213, 390–395 10.1007/s004250000494 11506361

[B80] Kovar D. R. Staiger C. J. Weaver E. A. McCurdy D. W. (2000). AtFim1 is an actin filament crosslinking protein from *Arabidopsis thaliana*. Plant J. 24, 625–636 10.1046/j.1365-313x.2000.00907.x 11123801

[B81] Krebs M. Beyhl D. Gorlich E. Al-Rasheid K. A. Marten I. Stierhof Y. D. Hedrich R. Schumacher K. (2010). Arabidopsis V-ATPase activity at the tonoplast is required for efficient nutrient storage but not for sodium accumulation. Proc. Natl. Acad. Sci. U.S.A. 107, 3251–3256 10.1073/pnas.0913035107 20133698 PMC2840351

[B82] Leckie C. P. Callow J. A. Green J. R. (1995). Reorganization of the endoplasmic reticulum in pea leaf epidermal cells infected by the powdery mildew fungus *Erysiphe pisi*. New Phytol. 131, 211–221

[B83] Lee B. S. Gluck S. L. Holliday L. S. (1999). Interaction between vacuolar H(+)-ATPase and microfilaments during osteoclast activation. J. Biol. Chem. 274, 29164–29171 10.1074/jbc.274.41.29164 10506172

[B84] Lemichez E. Wu Y. Sanchez J. P. Mettouchi A. Mathur J. Chua N. H. (2001). Inactivation of AtRac1 by abscisic acid is essential for stomatal closure. Genes Dev. 15, 1808–1816 10.1101/gad.900401 11459830 PMC312738

[B85] Li Y. Shen Y. Cai C. Zhong C. Zhu L. Yuan M. Ren H. (2010). The type II Arabidopsis formin14 interacts with microtubules and microfilaments to regulate cell division. Plant Cell 22, 2710–2726 10.1105/tpc.110.075507 20709814 PMC2947165

[B86] Liu B. Q. Jin L. Zhu L. Li J. Huang S. Yuan M. (2009). Phosphorylation of microtubule-associated protein SB401 from *Solanum berthaultii* regulates its effect on microtubules. J. Integr. Plant Biol. 51, 235–242 10.1111/j.1744-7909.2008.00797.x 19261066

[B87] Liu J. Seul U. Thompson R. (1997). Cloning and characterization of a pollen-specific cDNA encoding a glutamic-acid-rich protein (GARP) from potato *Solanum berthaultii*. Plant. Mol. Biol. 33, 291–300 10.1023/A:1005746713759 9037147

[B88] Liu K. Luan S. (1998). Voltage-dependent K+ channels as targets of osmosensing in guard cells. Plant Cell 10, 1957–1970 10.1105/tpc.10.11.1957 9811801 PMC143957

[B89] Lovy-Wheeler A. Wilsen K. L. Baskin T. I. Hepler P. K. (2005). Enhanced fixation reveals the apical cortical fringe of actin filaments as a consistent feature of the pollen tube. Planta 221, 95–104 10.1007/s00425-004-1423-2 15747143

[B90] Ma B. Qian D. Nan Q. Tan C. An L. Xiang Y. (2012). Arabidopsis vacuolar H+-ATPase (V-ATPase) B subunits are involved in actin cytoskeleton remodeling via binding to, bundling, and stabilizing F-actin. J. Biol. Chem. 287, 19008–19017 10.1074/jbc.M111.281873 22371505 PMC3365934

[B91] Ma B. Xiang Y. An L. (2011). Structural bases of physiological functions and roles of the vacuolar H(+)-ATPase. Cell. Signal. 23, 1244–1256 10.1016/j.cellsig.2011.03.003 21397012

[B92] Macrobbie E. A. Kurup S. (2007). Signalling mechanisms in the regulation of vacuolar ion release in guard cells. New Phytol. 175, 630–640 10.1111/j.1469-8137.2007.02131.x 17688580

[B93] Martiniere A. Gayral P. Hawes C. Runions J. (2011). Building bridges: formin1 of Arabidopsis forms a connection between the cell wall and the actin cytoskeleton. Plant J. 66, 354–365 10.1111/j.1365-313X.2011.04497.x 21241388

[B94] Michelot A. Berro J. Guerin C. Boujemaa-Paterski R. Staiger C. J. Martiel J. L. Blanchoin L. (2007). Actin-filament stochastic dynamics mediated by ADF/cofilin. Curr. Biol. 17, 825–833 10.1016/j.cub.2007.04.037 17493813

[B95] Michelot A. Derivery E. Paterski-Boujemaa R. Guerin C. Huang S. Parcy F. Staiger C. J. Blanchoin L. (2006). A novel mechanism for the formation of actin-filament bundles by a nonprocessive formin. Curr. Biol. 16, 1924–1930 10.1016/j.cub.2006.07.054 17027489

[B96] Michelot A. Guerin C. Huang S. Ingouff M. Richard S. Rodiuc N. Staiger C. J. Blanchoin L. (2005). The formin homology 1 domain modulates the actin nucleation and bundling activity of Arabidopsis FORMIN1. Plant Cell 17, 2296–2313 10.1105/tpc.105.030908 15994911 PMC1182490

[B97] Miller D. D. De Ruijter N. C. Bisseling T. Emons A. M. (1999). The role of actin in root hair morphogenesis: studies with lipochito-oligosaccharide as a growth stimulator and cytochalasin as an actin perturbing drug. Plant J. 17, 141–154

[B98] Moes D. Gatti S. Hoffmann C. Dieterle M. Moreau F. Neumann K. Schumacher M. Diederich M. Grill E. Shen W. H. Steinmetz A. Thomas C. (2012). A LIM domain protein from tobacco involved in actinbundling and histone gene transcription. Mol. Plant. [Epub ahead of print]. 10.1093/mp/SSS075 PMC3603003 22930731

[B99] Morley S. C. (2012). The actin-bundling protein L-plastin: a critical regulator of immune cell function. Int. J. Cell Biol. 2012, Article ID: 935173. 10.1155/2012/935173 22194750 PMC3238366

[B100] Mundel C. Baltz R. Eliasson A. Bronner R. Grass N. Krauter R. Evrard J. L. Steinmetz A. (2000). A LIM-domain protein from sunflower is localized to the cytoplasm and/or nucleus in a wide variety of tissues and is associated with the phragmoplast in dividing cells. Plant Mol. Biol. 42, 291–302 10.1023/A:1006333611189 10794529

[B101] Nelson N. (2003). A journey from mammals to yeast with vacuolar H+-ATPase (V-ATPase). J. Bioenerg. Biomembr. 35, 281–289 10.1023/A:1025768529677 14635774

[B102] Nick P. (2010). Probing the actin-auxin oscillator. Plant Signal. Behav. 5, 94–98 10.4161/psb.5.2.10337 20023411 PMC2884107

[B103] Nick P. Han M. J. An G. (2009). Auxin stimulates its own transport by shaping actin filaments. Plant Physiol. 151, 155–167 10.1104/pp.109.140111 19633235 PMC2736007

[B104] Nishi T. Forgac M. (2002). The vacuolar (H+)-ATPases–nature's most versatile proton pumps. Nat. Rev. Mol. Cell Biol. 3, 94–103 10.1038/nrm729 11836511

[B105] Oikawa K. Kasahara M. Kiyosue T. Kagawa T. Suetsugu N. Takahashi F. Kanegae T. Niwa Y. Kadota A. Wada M. (2003). Chloroplast unusual positioning1 is essential for proper chloroplast positioning. Plant Cell 15, 2805–2815 10.1105/tpc.016428 14615600 PMC282804

[B106] Oikawa K. Yamasato A. Kong S. G. Kasahara M. Nakai M. Takahashi F. Ogura Y. Kagawa T. Wada M. (2008). Chloroplast outer envelope protein CHUP1 is essential for chloroplast anchorage to the plasma membrane and chloroplast movement. Plant Physiol. 148, 829–842 10.1104/pp.108.123075 18715957 PMC2556824

[B107] Ono S. (2007). Mechanism of depolymerization and severing of actin filaments and its significance in cytoskeletal dynamics. Int. Rev. Cytol. 258, 1–82 10.1016/S0074-7696(07)58001-0 17338919

[B108] Opalski K. S. Schultheiss H. Kogel K. H. Huckelhoven R. (2005). The receptor-like MLO protein and the RAC/ROP family G-protein RACB modulate actin reorganization in barley attacked by the biotrophic powdery mildew fungus *Blumeria graminis* f.sp. hordei. Plant J. 41, 291–303 10.1111/j.1365-313X.2004.02292.x 15634205

[B109] Padmanaban S. Lin X. Perera I. Kawamura Y. Sze H. (2004). Differential expression of vacuolar H+-ATPase subunit c genes in tissues active in membrane trafficking and their roles in plant growth as revealed by RNAi. Plant Physiol. 134, 1514–1526 10.1104/pp.103.034025 15051861 PMC419827

[B110] Papuga J. Hoffmann C. Dieterle M. Moes D. Moreau F. Tholl S. Steinmetz A. Thomas C. (2010). Arabidopsis LIM proteins: a family of actin bundlers with distinct expression patterns and modes of regulation. Plant Cell 22, 3034–3052 10.1105/tpc.110.075960 20817848 PMC2965535

[B111] Paves H. Truve E. (2007). Myosin inhibitors block accumulation movement of chloroplasts in *Arabidopsis thaliana* leaf cells. Protoplasma 230, 165–169 10.1007/s00709-006-0230-y 17458631

[B112] Peremyslov V. V. Prokhnevsky A. I. Avisar D. Dolja V. V. (2008). Two class XI myosins function in organelle trafficking and root hair development in Arabidopsis. Plant Physiol. 146, 1109–1116 10.1104/pp.107.113654 18178669 PMC2259062

[B113] Petrasek J. Schwarzerova K. (2009). Actin and microtubule cytoskeleton interactions. Curr. Opin. Plant Biol. 12, 728–734 10.1016/j.pbi.2009.09.010 19854097

[B114] Pollard T. D. Blanchoin L. Mullins R. D. (2000). Molecular mechanisms controlling actin filament dynamics in nonmuscle cells. Annu. Rev. Biophys. Biomol. Struct. 29, 545–576 10.1146/annurev.biophys.29.1.545 10940259

[B115] Pollard T. D. Cooper J. A. (2009). Actin, a central player in cell shape and movement. Science 326, 1208–1212 10.1126/science.1175862 19965462 PMC3677050

[B116] Ruzicka D. R. Kandasamy M. K. McKinney E. C. Burgos-Rivera B. Meagher R. B. (2007). The ancient subclasses of Arabidopsis actin depolymerizing factor genes exhibit novel and differential expression. Plant J. 52, 460–472 10.1111/j.1365-313X.2007.03257.x 17877706

[B117] Samstag Y. Klemke M. (2007). Ectopic expression of L-plastin in human tumor cells: diagnostic and therapeutic implications. Adv. Enzyme Regul. 47, 118–126 10.1016/j.advenzreg.2006.12.008 17335876

[B118] Schmidt Von Braun S. Schleiff E. (2008a). The chloroplast outer membrane protein CHUP1 interacts with actin and profilin. Planta 227, 1151–1159 10.1007/s00425-007-0688-7 18193273

[B119] Schmidt Von Braun S. Schleiff E. (2008b). Moving the green: CHUP1 and chloroplast movement-an obvious relationship? Plant Signal. Behav. 3, 488–489 10.4161/psb.3.7.5683 19704495 PMC2634439

[B120] Schumacher K. Vafeados D. McCarthy M. Sze H. Wilkins T. Chory J. (1999). The Arabidopsis det3 mutant reveals a central role for the vacuolar H(+)-ATPase in plant growth and development. Genes Dev. 13, 3259–3270 10617574 10.1101/gad.13.24.3259 PMC317205

[B121] Sheahan M. B. Staiger C. J. Rose R. J. McCurdy D. W. (2004). A green fluorescent protein fusion to actin-binding domain 2 of Arabidopsis fimbrin highlights new features of a dynamic actin cytoskeleton in live plant cells. Plant Physiol. 136, 3968–3978 10.1104/pp.104.049411 15557099 PMC535829

[B122] Shinomiya H. (2012). Plastin family of actin-bundling proteins: its functions in leukocytes, neurons, intestines, and cancer. Int. J. Cell Biol. 2012, 213492 10.1155/2012/213492 22262972 PMC3259490

[B123] Showalter A. M. Keppler B. Lichtenberg J. Gu D. Welch L. R. (2010). A bioinformatics approach to the identification, classification, and analysis of hydroxyproline-rich glycoproteins. Plant Physiol. 153, 485–513 10.1104/pp.110.156554 20395450 PMC2879790

[B124] Skau C. T. Courson D. S. Bestul A. J. Winkelman J. D. Rock R. S. Sirotkin V. Kovar D. R. (2011). Actin filament bundling by fimbrin is important for endocytosis, cytokinesis, and polarization in fission yeast. J. Biol. Chem. 286, 26964–26977 10.1074/jbc.M111.239004 21642440 PMC3143655

[B125] Smertenko A. P. Deeks M. J. Hussey P. J. (2010). Strategies of actin reorganisation in plant cells. J. Cell Sci. 123, 3019–3028 10.1242/jcs.071126 20699356

[B126] Smith L. G. Oppenheimer D. G. (2005). Spatial control of cell expansion by the plant cytoskeleton. Annu. Rev. Cell Dev. Biol. 21, 271–295 10.1146/annurev.cellbio.21.122303.114901 16212496

[B127] Sparkes I. A. Teanby N. A. Hawes C. (2008). Truncated myosin XI tail fusions inhibit peroxisome, golgi, and mitochondrial movement in tobacco leaf epidermal cells: a genetic tool for the next generation. J. Exp. Bot. 59, 2499–2512 10.1093/jxb/ern114 18503043 PMC2423659

[B128] Staiger C. J. Blanchoin L. (2006). Actin dynamics: old friends with new stories. Curr. Opin. Plant Biol. 9, 554–562 10.1016/j.pbi.2006.09.013 17011229

[B129] Staiger C. J. Hussey P. J. (2004). Actin and actin-modulating proteins, in The Plant Cytoskeleton in Cell Differentiation and Development, ed P. J. Hussey (Oxford, UK: Blackwell Publishing), 32–80

[B130] Staiger C. J. Poulter N. S. Henty J. L. Franklin-Tong V. E. Blanchoin L. (2010). Regulation of actin dynamics by actin-binding proteins in pollen. J. Exp. Bot. 61, 1969–1986 10.1093/jxb/erq012 20159884

[B131] Staiger C. J. Sheahan M. B. Khurana P. Wang X. McCurdy D. W. Blanchoin L. (2009). Actin filament dynamics are dominated by rapid growth and severing activity in the Arabidopsis cortical array. J. Cell Biol. 184, 269–280 10.1111/j.1365-313X.2004.02292.x 19171759 PMC2654301

[B132] Staiger C. J. Yuan M. Valenta R. Shaw P. J. Warn R. M. Lloyd C. W. (1994). Microinjected profilin affects cytoplasmic streaming in plant cells by rapidly depolymerizing actin microfilaments. Curr. Biol. 4, 215–219 10.1016/S0960-9822(00)00050-6 7922326

[B133] Stevenson R. P. Veltman D. Machesky L. M. (2012). Actin-bundling proteins in cancer progression at a glance. J. Cell Sci. 125, 1073–1079 10.1242/jcs.093799 22492983

[B134] Suetsugu N. Yamada N. Kagawa T. Yonekura H. Uyeda T. Q. Kadota A. Wada M. (2010a). Two kinesin-like proteins mediate actin-based chloroplast movement in *Arabidopsis thaliana*. Proc. Natl. Acad. Sci. U.S.A. 107, 8860–8865 10.1073/pnas.0912773107 20418504 PMC2889332

[B135] Suetsugu N. Dolja V. V. Wada M. (2010b). Why have chloroplasts developed a unique motility system? Plant Signal. Behav. 5, 1190–1196 10.4161/psb.5.10.12802 20855973 PMC3115347

[B136] Suetsugu N. Wada M. (2007). Chloroplast photorelocation movement mediated by phototropin family proteins in green plants. Biol. Chem. 388, 927–935 10.1515/BC.2007.118 17696776

[B137] Szymanski D. B. (2005). Breaking the WAVE complex: the point of Arabidopsis trichomes. Curr. Opin. Plant Biol. 8, 103–112 10.1016/j.pbi.2004.11.004 15653407

[B138] Szymanski D. B. Cosgrove D. J. (2009). Dynamic coordination of cytoskeletal and cell wall systems during plant cell morphogenesis. Curr. Biol. 19, R800– R811 10.1016/j.cub.2009.07.056 19906582

[B139] Takemoto D. Jones D. A. Hardham A. R. (2006). Re-organization of the cytoskeleton and endoplasmic reticulum in the Arabidopsis pen1-1 mutant inoculated with the non-adapted powdery mildew pathogen, *Blumeria graminis* f. sp. hordei. Mol. Plant Pathol. 7, 553–563 10.1111/j.1364-3703.2006.00360.x 20507469

[B140] Tholl S. Moreau F. Hoffmann C. Arumugam K. Dieterle M. Moes D. Neumann K. Steinmetz A. Thomas C. (2011). Arabidopsis actin-depolymerizing factors (ADFs) 1 and 9 display antagonist activities. FEBS Lett. 585, 1821–1827 10.1016/j.febslet.2011.05.019 21570971

[B141] Thomas C. Hoffmann C. Dieterle M. Van Troys M. Ampe C. Steinmetz A. (2006). Tobacco WLIM1 is a novel F-actin binding protein involved in actin cytoskeleton remodeling. Plant Cell 18, 2194–2206 10.1105/tpc.106.040956 16905656 PMC1560925

[B142] Thomas C. Moreau F. Dieterle M. Hoffmann C. Gatti S. Hofmann C. Van Troys M. Ampe C. Steinmetz A. (2007). The LIM domains of WLIM1 define a new class of actin bundling modules. J. Biol. Chem. 282, 33599–33608 10.1074/jbc.M703691200 17827159

[B143] Thomas C. Tholl S. Moes D. Dieterle M. Papuga J. Moreau F. Steinmetz A. (2009). Actin bundling in plants. Cell. Motil. Cytoskeleton 66, 940–957 10.1002/cm.20389 19504571

[B144] Tominaga M. Yokota E. Vidali L. Sonobe S. Hepler P. K. Shimmen T. (2000). The role of plant villin in the organization of the actin cytoskeleton, cytoplasmic streaming and the architecture of the transvacuolar strand in root hair cells of Hydrocharis. Planta 210, 836–843 10.1007/s004250050687 10805457

[B145] Toth R. Gerding-Reimers C. Deeks M. J. Menninger S. Gallegos R. M. Tonaco I. A. Hubel K. Hussey P. J. Waldmann H. Coupland G. (2012). Prieurianin/endosidin 1 is an actin-stabilizing small molecule identified from a chemical genetic screen for circadian clock effectors in *Arabidopsis thaliana*. Plant J. 71, 338–352 10.1111/j.1365-313X.2012.04991.x 22409627

[B146] Van Damme D. Bouget F. Y. Van Poucke K. Inze D. Geelen D. (2004). Molecular dissection of plant cytokinesis and phragmoplast structure: a survey of GFP-tagged proteins. Plant J. 40, 386–398 10.1111/j.1365-313X.2004.02222.x 15469496

[B147] van der Honing H. S. Kieft H. Emons A. M. Ketelaar T. (2012). Arabidopsis VILLIN2 and VILLIN3 are required for the generation of thick actin filament bundles and for directional organ growth. Plant Physiol. 158, 1426–1438 10.1104/pp.111.192385 22209875 PMC3291277

[B148] Van Troys M. Vandekerckhove J. Ampe C. (2008a). Actin and actin-binding proteins in cancer progression and metastasis, in Actin-Binding Proteins and Disease, Protein Reviews, eds Dos Remedios C. G. Chhabra D. (New York, NY: Springer), 229–277

[B149] Van Troys M. Huyck L. Leyman S. Dhaese S. Vandekerkhove J. Ampe C. (2008b). Ins and outs of ADF/cofilin activity and regulation. Eur. J. Cell Biol. 87, 649–667 10.1016/j.ejcb.2008.04.001 18499298

[B150] Vicente-Manzanares M. Ma X. Adelstein R. S. Horwitz A. R. (2009). Non-muscle myosin II takes centre stage in cell adhesion and migration. Nat. Rev. Mol. Cell Biol. 10, 778–790 10.1038/nrm2786 19851336 PMC2834236

[B151] Vitavska O. Merzendorfer H. Wieczorek H. (2005). The V-ATPase subunit C binds to polymeric F-actin as well as to monomeric G-actin and induces cross-linking of actin filaments. J. Biol. Chem. 280, 1070–1076 10.1074/jbc.M406797200 15525650

[B152] Vitavska O. Wieczorek H. Merzendorfer H. (2003). A novel role for subunit C in mediating binding of the H+-V-ATPase to the actin cytoskeleton. J. Biol. Chem. 278, 18499–18505 10.1074/jbc.M212844200 12606563

[B153] Wang H. J. Wan A. R. Jauh G. Y. (2008). An actin binding protein, LlLIM1, mediates Ca and H regulation of actin dynamics in pollen tubes. Plant Physiol. 47, 1619–1636 10.1104/pp.108.118604 18480376 PMC2492651

[B154] Wang X. L. Gao X. Q. Wang X. C. (2011). Stochastic dynamics of actin filaments in guard cells regulating chloroplast localization during stomatal movement. Plant Cell Environ. 34, 1248–1257 10.1111/j.1365-3040.2011.02325.x 21443604

[B155] Wang Y. S. Motes C. M. Mohamalawari D. R. Blancaflor E. B. (2004). Green fluorescent protein fusions to Arabidopsis fimbrin 1 for spatio-temporal imaging of F-actin dynamics in roots. Cell. Motil. Cytoskeleton 59, 79–93 10.1002/cm.20024 15362112

[B155a] Way J. C. Chalfie M. (1988). mec-3, a homeobox-containing gene that specifies differentiation of the touch receptor neurons in *C. elegans* Cell 54, 5–16 10.1016/0092-8674(88)90174-2 2898300

[B156] Whippo C. W. Khurana P. Davis P. A. Deblasio S. L. Desloover D. Staiger C. J. Hangarter R. P. (2011). THRUMIN1 is a light-regulated actin-bundling protein involved in chloroplast motility. Curr. Biol. 21, 59–64 10.1016/j.cub.2010.11.059 21185188

[B157] Wightman R. Turner S. R. (2008). The roles of the cytoskeleton during cellulose deposition at the secondary cell wall. Plant J. 54, 794–805 10.1111/j.1365-313X.2008.03444.x 18266917

[B158] Winder S. J. Ayscough K. R. (2005). Actin-binding proteins. J. Cell Sci. 118, 651–654 10.1242/jcs.01670 15701920

[B159] Wu Y. Yan J. Zhang R. Qu X. Ren S. Chen N. Huang S. (2010). Arabidopsis FIMBRIN5, an actin bundling factor, is required for pollen germination and pollen tube growth. Plant Cell 22, 3745–3763 10.1105/tpc.110.080283 21098731 PMC3015131

[B160] Xu J. R. Staiger C. J. Hamer J. E. (1998). Inactivation of the mitogen-activated protein kinase Mps1 from the rice blast fungus prevents penetration of host cells but allows activation of plant defense responses. Proc. Natl. Acad. Sci. U.S.A. 95, 12713–12718 10.1073/pnas.95.21.12713 9770551 PMC22896

[B161] Xu T. Forgac M. (2001). Microtubules are involved in glucose-dependent dissociation of the yeast vacuolar [H+]-ATPase *in vitro*. J. Biol. Chem. 276, 24855–24861 10.1074/jbc.M100637200 11331282

[B162] Xue X. H. Guo C. Q. Du F. Lu Q. L. Zhang C. M. Ren H. Y. (2011). AtFH8 is involved in root development under effect of low-dose latrunculin B in dividing cells. Mol. Plant 4, 264–278 10.1093/mp/ssq085 21307369

[B163] Yang W. Ren S. Zhang X. Gao M. Ye S. Qi Y. Zheng Y. Wang J. Zeng L. Li Q. Huang S. He Z. (2011). BENT UPPERMOST INTERNODE1 encodes the class II formin FH5 crucial for actin organization and rice development. Plant Cell 23, 661–680 10.1105/tpc.110.081802 21307285 PMC3077787

[B164] Ye J. Xu M. (2012). Actin bundler PLIM2s are involved in the regulation of pollen development and tube growth in Arabidopsis. J. Plant Physiol. 169, 516–522 10.1016/j.jplph.2011.11.015 22209219

[B165] Ye J. Zheng Y. Yan A. Chen N. Wang Z. Huang S. Yang Z. (2009). Arabidopsis Formin3 directs the formation of actin cables and polarized growth in pollen tubes. Plant Cell 21, 3868–3884 10.1105/tpc.109.068700 20023198 PMC2814512

[B165a] Yi K. Guo C. Chen D. Zhao B. Yang B. Ren H. (2005). Cloning and functional characterization of a formin-like protein (AtFH8) from Arabidopsis. Plant Physiol. 138, 1071–1082 10.1104/pp.104.055665 15923338 PMC1150421

[B166] Yokota E. Takahara K. Shimmen Ki T. T. (1998). Actin-bundling protein isolated from pollen tubes of lily. Biochemical and immunocytochemical characterization. Plant Physiol. 116, 1421–1429 10.1104/pp.116.4.1421 9536060 PMC35050

[B167] Yokota E. Shimmen T. (1999). The 135-kDa actin-bundling protein from lily pollen tubes arranges F-actin into bundles with uniform polarity. Planta 209, 264–266 10.1007/s004250050631 10436230

[B168] Yokota E. Muto S. Shimmen T. (2000). Calcium-calmodulin suppresses the filamentous actin-binding activity of a 135-kilodalton actin-bundling protein isolated from lily pollen tubes. Plant Physiol. 123, 645–654 10.1104/pp.123.2.645 10859194 PMC59032

[B169] Yokota E. Vidali L. Tominaga M. Tahara H. Orii H. Morizane Y. Hepler P. K. Shimmen T. (2003). Plant 115-kDa actin-filament bundling protein, P-115-ABP, is a homologue of plant villin and is widely distributed in cells. Plant Cell Physiol. 44, 1088–1099 10.1093/pcp/pcg132 14581634

[B170] Yokota E. Tominaga M. Mabuchi I. Tsuji Y. Staiger C. J. Oiwa K. Shimmen T. (2005). Plant villin, Lily P-135-ABP, possesses G-actin binding activity and accelerates the polymerization and depolymerization of actin in a Ca2+-sensitive manner. Plant Cell Physiol. 46, 1690–1703 10.1093/pcp/pci185 16100394

[B171] Zhang H. Qu X. Bao C. Khurana P. Wang Q. Xie Y. Zheng Y. Chen N. Blanchoin L. Staiger C. J. Huang S. (2010). Arabidopsis VILLIN5, an actin filament bundling and severing protein, is necessary for normal pollen tube growth. Plant Cell 22, 2749–2767 10.1105/tpc.110.076257 20807879 PMC2947167

[B172] Zhang W. Zhao Y. Guo Y. Ye K. (2012). Plant actin-binding protein SCAB1 is dimeric actin cross-linker with atypical pleckstrin homology domain. J. Biol. Chem. 287, 11981–11990 10.1074/jbc.M111.338525 22356912 PMC3320945

[B174] Zhang Z. Zhang Y. Tan H. Wang Y. Li G. Liang W. Yuan Z. Hu J. Ren H. Zhang D. (2011a). RICE MORPHOLOGY DETERMINANT encodes the type II formin FH5 and regulates rice morphogenesis. Plant Cell 23, 681–700 10.1105/tpc.110.081349 21307283 PMC3077795

[B173] Zhang Y. Xiao Y. Du F. Cao L. Dong H. Ren H. (2011b). Arabidopsis VILLIN4 is involved in root hair growth through regulating actin organization in a Ca(2+)-dependent manner. New Phytol. 190, 667–682 10.1111/j.1469-8137.2010.03632.x 21275995

[B175] Zhao Y. Zhao S. Mao T. Qu X. Cao W. Zhang L. Zhang W. He L. Li S. Ren S. Zhao J. Zhu G. Huang S. Ye K. Yuan M. Guo Y. (2011). The plant-specific actin binding protein SCAB1 stabilizes actin filaments and regulates stomatal movement in Arabidopsis. Plant Cell 23, 2314–2330 10.1105/tpc.111.086546 21719691 PMC3160031

[B176] Zimniak L. Dittrich P. Gogarten J. P. Kibak H. Taiz L. (1988). The cDNA sequence of the 69-kDa subunit of the carrot vacuolar H+-ATPase. Homology to the beta-chain of F0F1-ATPases. J. Biol. Chem. 263, 9102–9112 2897965

[B177] Zuo J. Jiang J. Chen S. H. Vergara S. Gong Y. Xue J. Huang H. Kaku M. Holliday L. S. (2006). Actin binding activity of subunit B of vacuolar H+-ATPase is involved in its targeting to ruffled membranes of osteoclasts. J. Bone Miner Res. 21, 714–721 10.1359/jbmr.060201 16734386

[B178] Zuo J. Vergara S. Kohno S. Holliday L. S. (2008). Biochemical and functional characterization of the actin-binding activity of the B subunit of yeast vacuolar H+-ATPase. J. Exp. Biol. 211, 1102–1108 10.1242/jeb.013672 18344484

